# Greenness Evaluation of Piracetam Detection Through Spectrofluorimetric Method With Shilajit‐Derived Metal Oxide Nanosensors

**DOI:** 10.1155/ianc/6962798

**Published:** 2026-02-08

**Authors:** Azaa F. Al-Shalawi, Amal M. Al-Mohaimeed, Nawal A. Alarfaj, Maha F. El-Tohamy

**Affiliations:** ^1^ Department of Chemistry, College of Science, King Saud University, P.O. Box 22452, Riyadh, 11495, Saudi Arabia, ksu.edu.sa

**Keywords:** environmental sustainability, fluorescence sensing, green chemistry, metal oxide nanoparticles, piracetam, Shilajit

## Abstract

Piracetam (PRM) is a nootropic commonly used to improve cognitive function, memory, and learning ability. This method introduces a new spectrofluorimetric strategy for the identification of PRM, using metal oxide nanomaterials from Shilajit extract in a micellar medium. The technique is based on a unique fluorescent platform of aluminum oxide and nickel oxide nanoparticles (NPs) combined with sodium dodecyl sulfate (SDS). The metal oxide NPs were prepared by an environmentally friendly synthesis approach, using Shilajit extract as a dual‐function agent for reduction and stabilization. Their morphology, size, and structural properties were comprehensively analyzed using a range of spectroscopic and microscopic methods. The innovative technique utilizes the unique fluorescence properties of alumina and NiO NPs in the presence of SDS to detect PRM with remarkable sensitivity and selectivity. This method enables high‐precision measurements over a wide calibration range of 0.5–10 and 0.2–14 μg/mL for the two metal oxides, respectively. With PRM recoveries of 99.07% ± 0.65% and 99.60% ± 0.37%, the method has excellent accuracy and reliability. Medium precision was used to ensure that the method meets stringent precision standards. In addition, the environmentally friendly approach of using Shilajit extract for the sustainable synthesis of metal oxides reduces the impact on the environment while maintaining excellent analytical performance, as confirmed by an environmental impact assessment.

## 1. Introduction

Alzheimer’s disease is a chronic neurological disorder that progressively impairs memory, cognitive function, and behavior. As the most common cause of dementia, it leads to a steady deterioration of mental abilities and eventually impairs everyday tasks and personal autonomy. Early symptoms often include forgetfulness, confusion, and difficulty solving problems or speaking. As the disease progresses, severe memory loss, disorientation, and changes in mood or behavior can occur [1].

**FIGURE 1:**
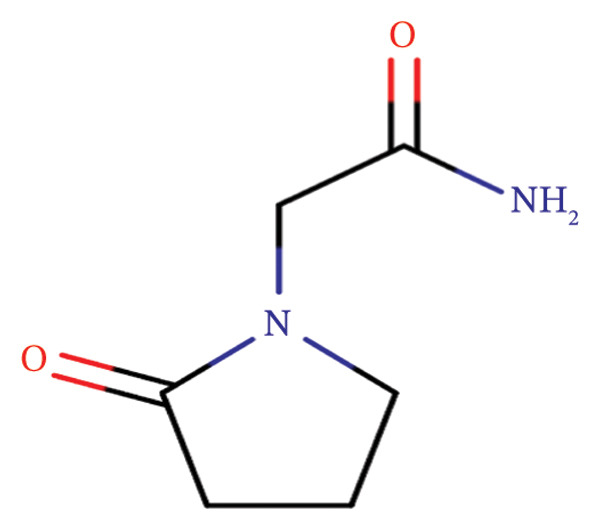
Piracetam chemical structure.

Piracetam (PRM) is sometimes considered a potential treatment for Alzheimer’s disease due to its cognition‐enhancing properties (Figure 1). As a nootropic, it is thought to improve memory, learning, and general brain function by affecting neurotransmitter systems, particularly acetylcholine [2]. Some studies suggest that PRM may help with mild cognitive impairment and slow the progression of Alzheimer’s symptoms [3].

Published studies have investigated a range of analytical techniques for the detection of PRM in different types of samples. These include chromatographic approaches [4–7], spectrophotometric analyses [8], voltammetric methods [9], and spectrofluorimetric methods [10].

Chromatographic, spectrophotometric, and electrochemical methods for the determination of PRM have certain disadvantages that limit their wide application. Chromatographic methods, although highly accurate, are often time‐consuming, require complex instrumentation, and involve the use of solvents, which can be costly and environmentally harmful [11]. Spectrophotometry, although relatively simple, can be interfered with by other substances in complex samples, reducing its specificity and accuracy [12]. Electrochemical methods, while offering high sensitivity, can be affected by electrode fouling and require careful calibration to ensure reliable results [13].

In contrast, fluorescence sensing, especially using novel metal oxide nanoparticles (NPs), offers a significant advantage as it provides high sensitivity and selectivity with a more environmentally friendly approach. This method avoids hazardous reagents and laborious sample preparation, providing a more environmentally friendly and potentially more economical option for drug analysis, without compromising analytical efficiency [14].

Shilajit has long been considered an important substance in traditional medicine, offering numerous health benefits, including increasing energy, improving cognitive function, and promoting general well‐being [15]. It is a complex compound rich in minerals, fulvic acid, and various bioactive phytochemicals that contribute to its medicinal properties. The major phytochemicals in Shilajit include phenolic compounds, flavonoids, alkaloids, and diterpenes, which have antioxidant, anti‐inflammatory, and antimicrobial properties [16]. In the synthesis of green NPs, these plant‐derived compounds can be used as both reducing and capping agents for metal oxide NPs [17].

In recent years, numerous reviews on the green synthesis of NPs have highlighted the promising potential of these methods for large‐scale production of metal oxides [18, 19]. Growing interest has been directed toward expanding the variety of plant extracts used in the synthesis of metal oxides [20–22]. This interest stems from the advantages plant‐based synthesis offers, such as avoiding the use of toxic chemicals, promoting environmental sustainability, and offering a more cost‐effective alternative to conventional methods.

Metal oxide NPs (such as alumina and NiO) are crucial for the measurement of fluorescence. The fluorescence behavior of these materials is influenced by their nanoscale dimensions, with quantum confinement effects altering their emission characteristics and enhancing their luminescence [23]. Alumina and NiO NPs have properties that enable their effective use in fluorescence‐based sensing. The high quantum yield and the modifiability of the surface contribute to their precision in the detection of pharmaceutical compounds [24–26].

This research paper presents a new method for the detection of PRM by a spectrofluorimetric technique using metal oxide NPs formed from Shilajit extract. Shilajit extract was selected for the synthesis of metal oxide NPs due to its rich natural composition of bioactive compounds, including humic acids, fulvic acids, and various minerals, which act as effective reducing and stabilizing agents in green synthesis processes. This environmentally friendly approach utilizes the inherent biochemical properties of Shilajit to produce NPs in a sustainable, nontoxic manner, avoiding harmful chemicals typically used in conventional synthesis. In addition, the use of Shilajit‐derived NPs enhances biocompatibility and provides unique surface properties that improve the sensitivity and selectivity of spectrofluorimetric detection of PRM, which is in line with the study’s goal of developing a more environmentally friendly and safer analytical method.

In addition, the study emphasizes sustainability by applying a green chemistry approach that minimizes the use of toxic chemicals and makes the process more environmentally friendly. The integration of Shilajit, a natural substance with rich bioactive compounds, not only improves analytical performance, but also contributes to the growing trend of utilizing natural materials for advanced nanotechnology applications. This combination of high sensitivity, selectivity, and environmental friendliness makes the study a significant contribution to both analytical chemistry and environmental sustainability.

## 2. Methodology

### 2.1. Materials

Pure PRM was purchased from Merck (New Jersey, United States), and Piracetam Al® 800 mg tablets were purchased from El‐Nahdi Pharmacy (Riyadh, Saudi Arabia). Other materials, such as aluminum nitrate nonahydrate, methanol (99.0%), acetonitrile (99.9%), and ethanol (97.0%), were provided by Sigma‐Aldrich (Hamburg, Germany). Sodium dodecyl sulfate (SDS, 99.5%), sodium hydroxide (97.0%), sodium dihydrogen phosphate (98.0%), boric acid (99.8%), sodium tetraborate (99.9%), sodium acetate (99.0%), Triton X‐100 (99.0%), and cetylpyridinium chloride (CPC, 99.9%) were purchased from BDH (Poole, UK). Shilajit resin was sourced from markets, in Riyadh, Saudi Arabia.

### 2.2. Instrumentation

The instruments used in this study were spectrofluorometer and spectrophotometer (Shimadzu‐ RF‐5301 and Shimadzu‐2600i, Kyoto, Japan), pH meter (Metrohm‐744, Herisau, Switzerland), a Perkin–Elmer spectrometer (Perkin–Elmer, Massachusetts, USA), a diffractometer (Shimadzu‐6000, Kyoto, Japan), scanning and transmission electron microscopes (SEM and TEM, JEOL Ltd., Tokyo, Japan), and energy‐dispersive X‐ray spectroscopy (EDX, Tokyo, Japan).

### 2.3. Preparation of Shilajit Extract

Approximately 100 mL of distilled water was added to 5 g of crude Shilajit powder and heated at 60°C for 2 h with magnetic stirring for 15 min. The mixture was centrifuged at 6000 rpm for 10 min. After separation, the clear supernatant was placed in a drying oven at 70°C and concentrated until the remaining moisture content was reduced to about 5%–10% [27].

### 2.4. Preparation of Nanomaterials Using Shilajit Extract

The green synthesis of alumina NPs and NiO NPs was carried out separately. For each synthesis, 20 mL of Shilajit extract was mixed with 100 mL of aluminum nitrate and nickel sulfate, respectively, which served as precursors for the respective metal oxide NPs. The mixture was heated to 80°C for 30 min with magnetic stirring. A few drops of a 2.0 mol/L sodium hydroxide solution were then added until a yellowish (for alumina NPs) or greenish (for NiO NPs) precipitate formed. The resulting precipitate was rinsed three times, first with ethanol and then with deionized water, before being filtered with Whatman filter paper No. 1. The NPs were then placed in an oven and dried at 100°C for 6 h.

For the calcination of NiO NPs, the dried precipitate is usually placed in a muffle furnace or a high‐temperature furnace at 400°C for 3 h to ensure complete removal of organic residues and obtain well‐crystallized metal oxide NPs. Once the metal oxide NPs are calcined, they are transferred to an airtight container and stored for later analysis and applications [26].

### 2.5. Preparation of Analytical Reagents

To prepare the acetate buffer (0.1 M, pH range 3.0–5.6), equal volumes of 0.1 M solutions of acetic acid and sodium acetate were combined in 1 L of deionized water, and the pH adjusted to the target value. For the phosphate buffer (0.2 M, pH 5.8–8.0), solutions of 0.2 M sodium dihydrogen phosphate and 0.2 M sodium hydroxide were prepared and then mixed in varying ratios to achieve the desired pH. The borate buffer (0.1 M, pH range 8.0–11.0) was prepared by mixing 500 mL of 0.1 M boric acid with 500 mL of 0.1 M sodium tetraborate, and the pH was fine‐tuned by adjusting the ratio of the two solutions.

### 2.6. Preparation of Standard Solution

A PRM stock solution with a concentration of 100 μg/mL was prepared by dissolving 10 mg PRM in 100 mL of distilled water. From this stock solution, a series of diluted solutions were prepared with the same solvent to obtain the desired concentrations for analysis. The concentration ranges for the fluorescent systems SDS‐alumina NPs and SDS‐NiO NPs were 0.5–10 μg/mL and 0.2–14.0 μg/mL, respectively. To facilitate future analyses, the samples were stored in sterile, airtight containers.

### 2.7. General Spectrofluorimetric Method

Fluorescence systems based on SDS‐alumina NPs and SDS‐NiO NPs were used for the determination of PRM at room temperature. The presence of PRM was determined by quantification of both the pure substance and the tablet‐extracted samples in 10‐mL volumetric flasks. Fluorescence intensity (FI) was measured at *λ*_ex_/*λ*_em_ of 350/400 nm. The analysis was performed with a mixture containing 2.0 mL alumina NPs or 1.5 mL NiO NPs, 0.2 mL SDS, and 2.0 mL acetate buffer (0.1 M, pH 4). A calibration curve was constructed to verify the linear response of the method, and a regression equation was used to quantify the unknown concentration of the drug sample.

## 3. Results and Discussion

### 3.1. Confirmation of Metal Oxide NPs

Confirmation of metal oxide NPs using UV–vis analysis is crucial as it provides valuable information about the optical properties of the NPs, such as absorption peaks, which can help confirm synthesis and stability. The UV–vis spectra can indicate the presence of surface plasmon resonance (SPR) bands that are characteristic of certain NPs, especially metal oxides [28].

The UV–vis spectrum of Shilajit typically shows absorption peaks, which may be due to the presence of various bioactive substances, including phenolic compounds, flavonoids, and other organic molecules. The absorption peak at 308 nm is often associated with the presence of aromatic compounds or conjugated double bonds that absorb light in this wavelength range. In Shilajit, this peak could indicate the presence of these phytochemicals. The absorbance at 308 nm could also be related to the characteristic functional groups in Shilajit that contribute to its bioactivity, such as the carbonyl or hydroxyl groups in its complex mixture of compounds [29].

The optical properties of alumina NPs were investigated at room temperature in the *λ*_max_ range of 200–600 nm. The UV–Vis spectrum of alumina NPs synthesized with Shilajit extract typically shows a band at 290 nm, which is due to the presence of certain organic molecules. The peak at 290 nm is probably related to the aromatic compounds, such as phenols and flavonoids, present in the Shilajit extract. These molecules have conjugated π‐electron systems that allow them to absorb ultraviolet light, especially at 290 nm. The Shilajit extract plays a dual role in the ecofriendly synthesis of alumina NPs by acting both as a reducing agent, helping to reduce metal ions into their nanoparticulate form, and as a stabilizing agent, preventing NP aggregation [28]. The peak at 290 nm in the UV–Vis spectrum confirms the effective interaction between the extract and the aluminum precursor, and it may also reflect the presence of surface functionalized groups on the alumina NPs. This characteristic absorption can be used to ensure the synthesis of the nanomaterials and their stability and at the same time provides information about the involvement of the bioactive compounds of Shilajit in the synthesis process of the NPs.

**FIGURE 2:**
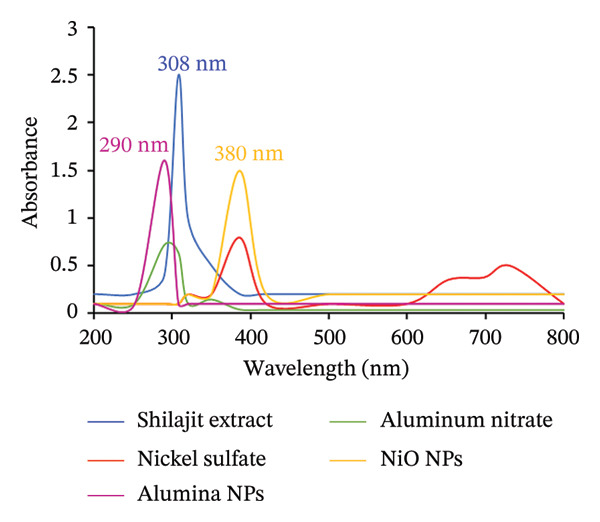
UV–vis spectra of Shilajit extract, aluminum nitrate, nickel sulfate, alumina NPs, and NiO NPs measured in the range 200–800 nm.

The UV–Vis spectrum of NiO NPs prepared with Shilajit extract generally shows distinct absorption bands, related to the electronic transitions within the NiO and its interaction with the components of the Shilajit extract. In the UV–vis spectrum, the NiO NPs generally show an absorption peak at 380 nm in the 300–400 nm range, which corresponds to the charge transfer transitions between the metal and oxygen in the NP structure. This absorption band is associated with NiO and correlates in particular with the interband transition and SPR phenomena (Figure 2). When synthesized with Shilajit extract, the UV–Vis spectrum may also show some additional features resulting from the bioactive compounds in Shilajit, such as phenolic compounds, flavonoids, and other organic molecules, that can absorb light in the UV region [30]. The band gap of NPs can be calculated directly from UV–vis absorption spectra by applying the Tauc Equation [31]:

(1) Eg=hCλ.

The parameters are represented as Eg (band gap energy), *h* (Planck’s constant, 6.626 × 10^−34^ J·s), C (speed of light, 2.99 × 10^8^ m/s), and *λ* (wavelength of absorption). The energy conversion factor used is 1 eV = 1.6 × 10^−19^ J. Based on calculations, the band gap energies for alumina and NiO NPs were determined to be 4.28 and 3.26 eV, respectively. These values are crucial for analyzing the optical and electronic characteristics of the synthesized nanomaterials.

FTIR analysis analyzes how molecules absorb infrared radiation at specific wavelengths, creating a unique spectral pattern that can be used to verify the chemical structure and composition of the material. In the FTIR spectrum of Shilajit, a broad and strong peak corresponding to the O‐H stretching vibrations can be seen at 3417 cm^−1^, indicating the presence of alcohol or phenolic functional groups. This indicates that Shilajit contains hydroxyl groups, which are crucial for its biological activity. The peak at 2924 cm^−1^ corresponds to the C‐H stretching vibrations, indicating aliphatic hydrocarbons, which can be associated with the lipid or fatty acid content of the extract. The subsequent peak at 2376 cm^−1^ could refer to C ≡ C or C ≡ N stretching vibrations, indicating the presence of alkynes or nitriles, which may have various bioactive properties. The peak at 1651 cm^−1^ is significant for C=O stretching, which is often associated with carbonyl compounds, which may include ketones or carboxylic acids.

**FIGURE 3:**
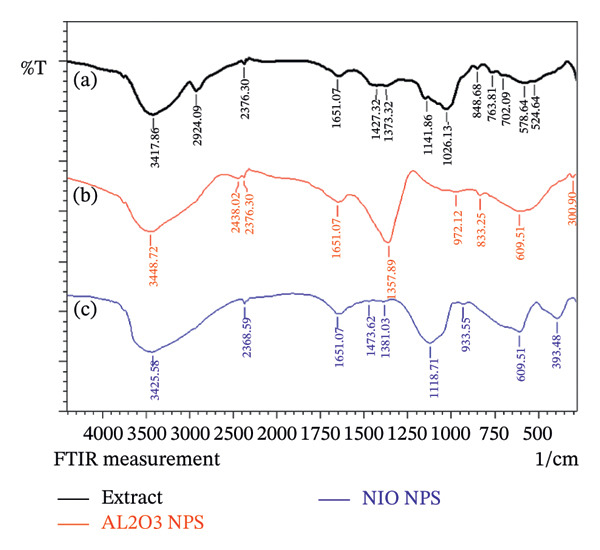
FTIR analysis of (a) Shilajit extract, (b) alumina NPs, and (c) NiO NPs.

This indicates that Shilajit contains bioactive compounds, such as fulvic acid or humic substances, that contribute to its therapeutic effect. The peak at 1427 cm^−1^ indicates C‐H bending vibrations that can be associated with the presence of CH_2_ groups, further emphasizing the complex mixture of organic compounds in shilajit. A peak observed at 1373 cm^−1^ is typically associated with C‐H bending vibrations of methyl groups, indicating the presence of aliphatic compounds. The peak at 1141 cm^−1^ relates to C‐O stretching vibrations that can be attributed to alcohols, ethers, or carbohydrates, suggesting that Shilajit contains sugars or polysaccharides that may enhance its health benefits. The peak at 1026 cm^−1^ can be attributed to C‐O‐C stretching, typically found in glycosides or polysaccharides, further supporting the idea of a complex carbohydrate structure in the extract. The peak at 748 cm^−1^ is likely due to out‐of‐plane C‐H bending vibrations, often associated with aromatic compounds, suggesting the presence of phenolic structures. Finally, the peak at 524 cm^−1^ could indicate metal‐O vibrations or other functional groups associated with minerals that are critical to the overall effect of Shilajit (Figure 3(a)).

In the FTIR spectrum of alumina NPs, the absorption band around 3448 cm^−1^ is generally attributed to O‐H stretching, indicating the presence of hydroxyl functions. This is often associated with the surface hydroxyl groups on the alumina NPs, which may result from the interaction of the NPs with moisture or from the functional groups in the Shilajit extract that help stabilize the NPs. The peak at 2438 cm^−1^ may reflect the presence of C ≡ N stretching vibrations, which may indicate nitriles or other compounds in the extract that have been incorporated into the alumina structure during synthesis. The peak at 2376 cm^−1^ may also indicate the presence of functional groups associated with alkynes or other organic constituents of Shilajit, suggesting the organic nature of the synthesis process. At 1651 cm^−1^, a peak corresponding to the C=O stretching vibrations is typically observed, which may indicate the presence of carbonyl groups. These groups are often associated with organic acids or other compounds in the Shilajit that help stabilize and functionalize the alumina NPs. The peak at 1357 cm^−1^ is related to C‐H bending vibrations, which are often associated with methyl or methylene groups that may originate from organic residues in the Shilajit extract that adhere to the NPs. This indicates that organic compounds contribute to changing the surface properties of the alumina NPs. The absorption band at 972 cm^−1^ is typically associated with Al‐O stretching vibrations, confirming the formation of alumina and supporting the identification of the synthesized material. In addition, the peak observed at 833 cm^−1^ is likely due to out‐of‐plane C‐H bending in aromatic structures, indicating the presence of phenolic components of Shilajit, which may improve the stability and functionality of the NPs. Finally, the peak at 609 cm^−1^ is indicative of metal‐oxygen bonding, particularly related to the Al‐O stretch, which is critical for confirming the formation of aluminum oxide as well as the structural integrity of the NPs (Figure 3(b)).

FTIR analysis of NiO NPs synthesized from shilajit reveals several important peaks that provide insight into both the structure of the NPs and the organic constituents of Shilajit. The broad absorption band at 3425 cm^−1^ is attributed to O‐H stretching vibrations, indicating the presence of hydroxyl groups, likely originating from water molecules or organic functions in Shilajit. The band at 2368 cm^−1^ could be due to adsorbed carbon dioxide, a typical environmental pollutant. Meanwhile, the peak at 1651 cm^−1^ is associated with C=O stretching, suggesting the presence of carbonyl or carboxyl groups and emphasizing the organic composition of Shilajit. The peaks at 1473 and 1381 cm^−1^ are associated with C‐H bending vibrations, which are typical of aliphatic groups in organic material. The peak at 1118 cm^−1^ indicates C‐O stretching, which is indicative of ether or alcohol functional groups. The peaks at 933, 609, and 393 cm^−1^ indicate Ni‐O bond stretching and confirm the successful formation of NiO NPs (Figure 3(c)).

X‐ray diffraction (XRD) analysis was performed using a diffractometer (Shimadzu‐6000, Kyoto, Japan), with Cu Kα radiation (wavelength 1.5406 Å) as the source, under operating conditions of 40 kV and 30 mA, scanning over a 2*θ* range of 20°–90°, with a step size of 0.05° and a scan rate of 5°/min. The XRD pattern of the alumina NP sample showed the presence of symmetrical, spherical crystallites arranged in a face‐centered cubic (FCC) structure (Figure 4(a)). The analysis showed several peaks corresponding to crystallographic planes at 32.5° (2 2 0), 35.1° (3 1 1), 38.7° (2 2 2), 46.5° (4 0 0), 62.4° (4 2 2), 67.2° (4 4 0), and 78.4° (6 2 0). These observed peaks were consistent with the standard JCPDS card‐79‐1558 [32]. Similarly, the NiO NP sample was examined by XRD, which revealed diffraction peaks at 37.20° (1 1 1), 43.30° (2 0 0), 62.87° (2 2 0), and 75.20° (3 1 1), corresponding to the crystal planes. These diffraction peaks matched well with the FCC structure of NiO, as shown by the standard JCPDS card No. 65‐2901 [33], both in terms of peak positions and relative intensities (Figure 4(b)). The Debye–Scherrer equation [34] was used to estimate the average grain size of each nanomaterial synthesized via the green method.

(2) D=0.94λβCos θ.

FIGURE 4XRD patterns of (a) alumina NPs and (b) NiO NPs synthesized by Shilajit extract in comparison with standard cards.

**Figure:**
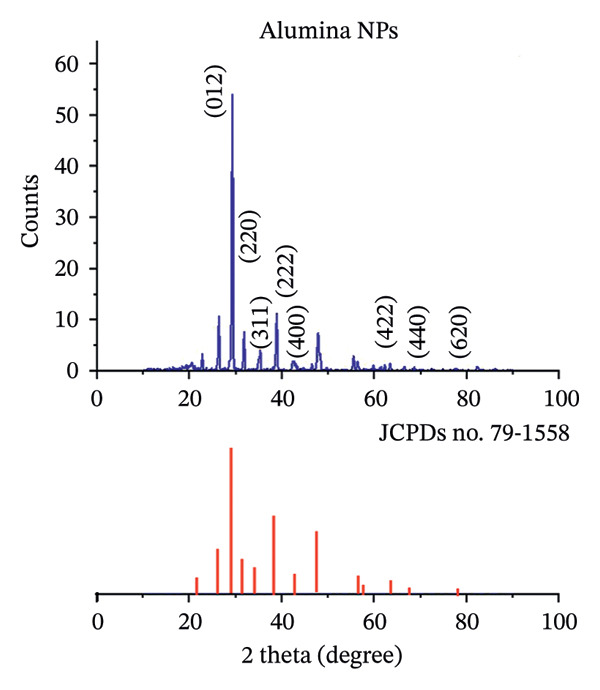
(a)

**Figure:**
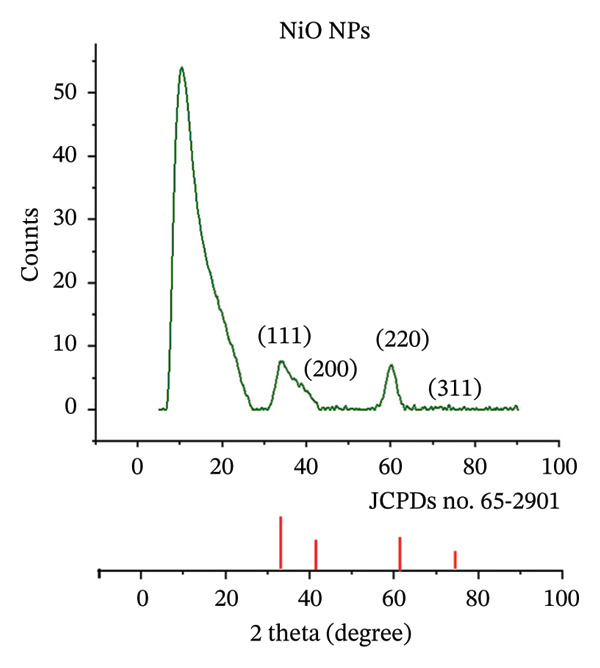
(b)

In this equation, *D* denotes the average crystallite size, *λ* is the X‐ray wavelength (1.54056 Å for Cu Kα radiation), *β* represents the full width at half maximum (FWHM) of the diffraction peak, and *θ* is the Bragg angle. Based on this, the estimated grain sizes for alumina and NiO NPs were found to be 18.73 ± 1.45 nm and 18.75 ± 1.37 nm, respectively.

The crystalline % can be estimated from the following equation [35]:

(3) CrI %=Icryst−IamIcryst×100,

where *I*_cryst_ = maximum intensity of the crystalline peak (sharp peak usually around 2*θ* = 20°–30° or depending on the material). *I*_am_ = minimum intensity at amorphous background (the valley between peaks). For the XRD pattern of alumina NPs, the main crystalline peak *I*_cryst_ appears around 2*θ* ≈ 20–30 and 35°–45°; there is no significant broad hump meaning the amorphous contribution is very small. This indicates the samples of alumina NPs are highly crystalline with crystalline % around 85%–90%. For NiO NPs, the main crystalline peak *I*_cryst_ appears around 2*θ* ≈ 35°–40° with an intensity of about 160 a.u. The amorphous background *I*_am_ is around 2*θ* ≈ 20° with intensity of about 40°a.u. The crystallinity % = 75%.

Based on the crystallinity values given, the estimated porosity of the NPs can be derived by assuming that the amorphous fraction contributes to the overall porosity. For NiO NPs with a crystallinity of 35%–45%, the corresponding amorphous fraction is 55%–65%, suggesting an estimated porosity in the range of 55%–65%. In contrast, aluminum oxide NPs have a much higher crystallinity of 85%–90%, indicating a lower amorphous fraction of 10%–15% and thus an estimated porosity of 10%–15%.

(4) Porosity %−1−crystallinity fraction×100.

To calculate the specific surface area for the form alumina and NiO NPs, the following equation can be used [36]:

(5) SSA=6D.ρ,

where SSA = specific surface area (m^2^/g), *D* = average particle size or crystallite size (m), and *ρ* = density of the material (g/cm^3^ or kg/m^3^). The alumina (Al_2_O_3_) SSA ≈ 81.07 m^2^/g and NiO ≈ 47.98 m^2^/g.

The SEM makes an important contribution to the detailed characterization of NPs by providing high‐resolution images that allow a comprehensive analysis of their shape and size. In this study, the SEM was used to evaluate the morphology of NPs. Spherical structures with an average size of 71.16 ± 9.61 nm for alumina NPs and 77.63 ± 7.83 nm for NiO NPs were observed, as shown in Figures 5(a) and 5(c). Detailed images were obtained using SEM, which confirmed the spherical morphology of the particles while allowing the investigation of important surface features, such as dispersion properties and aggregation patterns. The pseudo‐3D effect created by the SEM further improves the visibility of the surface contours and provides valuable insights into the spatial arrangement of the NPs. Therefore, SEM examination is essential to confirm the structure and surface properties of NPs with high precision.

FIGURE 5(a, c) SEM images and (b, d) AFM 3D images; (e, f) particle size distribution of alumina NPs and NiO NPs synthesized by Shilajit extract.

**Figure:**
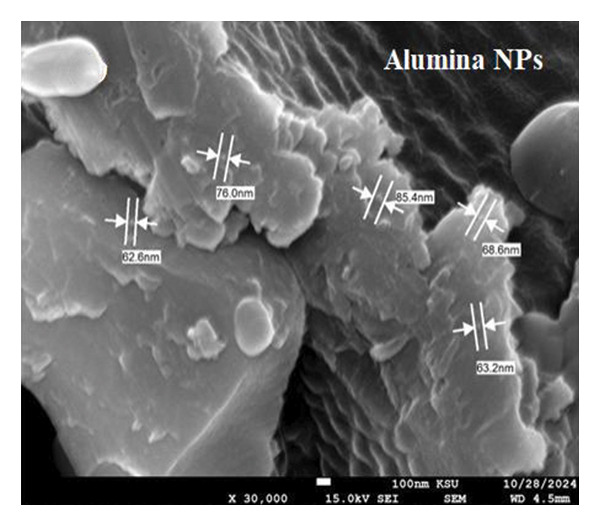
(a)

**Figure:**
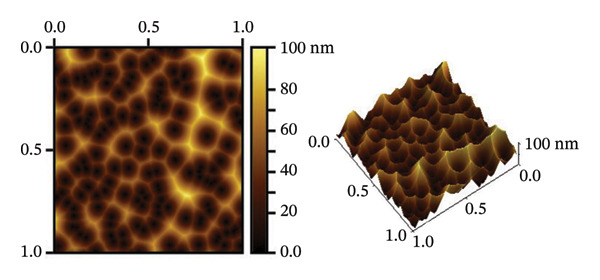
(b)

**Figure:**
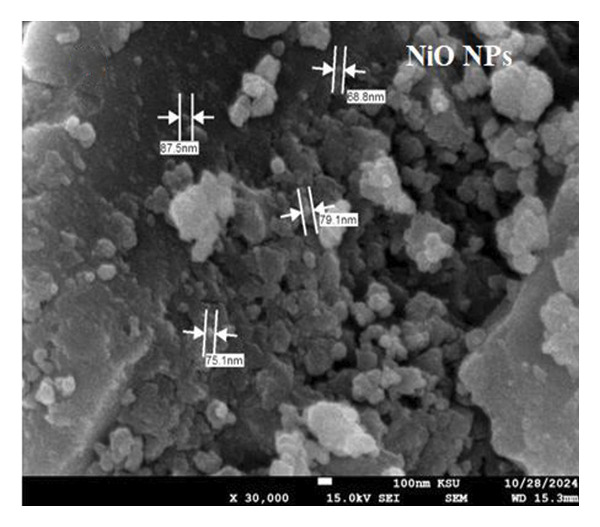
(c)

**Figure:**
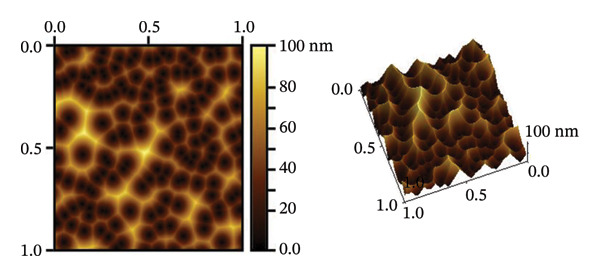
(d)

**Figure:**
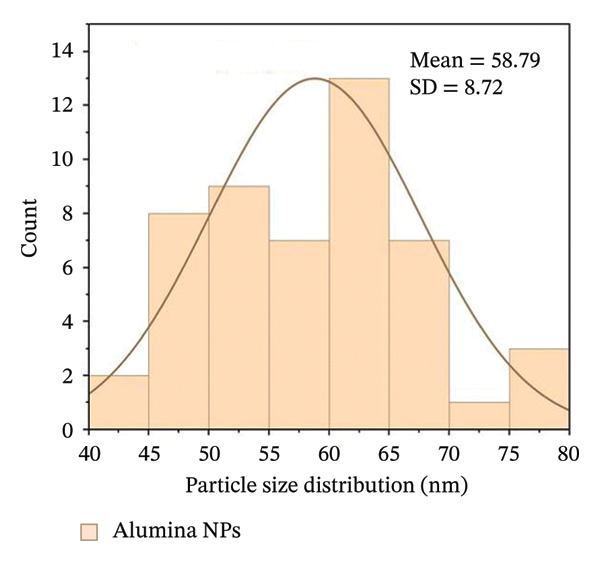
(e)

**Figure:**
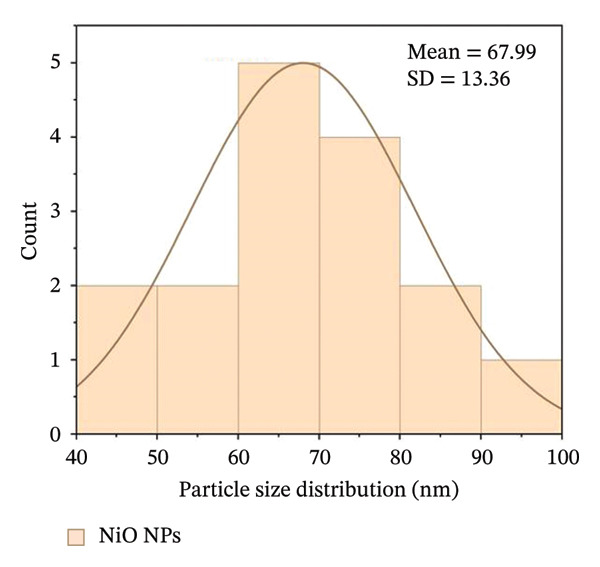
(f)

Atomic force microscopy (AFM) is an indispensable tool for the detailed analysis of the properties of NPs. It provides high‐resolution, three‐dimensional topographical data that are crucial for understanding particle morphology.

Unlike SEM, which provides two‐dimensional images, AFM provides 3D surface imaging and allows accurate assessment of particle height and surface texture, without the need for conductive coatings. Figures 5(b) and 5(d) show how AFM complements other characterization techniques by providing detailed topographical information, enabling a comprehensive understanding of NP properties. While SEM effectively captures overall morphology and size, high‐resolution AFM imaging reveals subtle surface features, such as roughness and texture, that are critical for evaluating NP performance and functionality.

Figures 5(e) and 5(f) show the histograms of the particle size distribution of the alumina and NiO NPs synthesized with Shilajit extract based on SEM/AFM analysis. For the alumina NPs, the particle sizes are mostly between ∼45 and 75 nm with a mean size of 58.79 ± 8.72 nm, indicating a more uniform size distribution. For the NiO NPs, the particle size distribution is more in the range of ∼60–100 nm with a mean size of 67.99 ± 13.36 nm, indicating greater polydispersity compared to the alumina NPs. The fitted Gaussian curves show that both materials follow approximately normal size distribution patterns.

The TEM images show the morphology of NiO and alumina NPs synthesized using Shilajit extract, a natural organic reducing and stabilizing agent. In Figure 6(a), the NiO NPs appear as aggregated, nearly spherical particles with good contrast, indicating crystallinity and relatively uniform size. In Figure 6(b), the alumina NPs display a more densely packed and uniform distribution, also with mostly spherical morphology. Both types of NPs exhibit sizes consistent with the 60–70 nm range, confirming nanoscale synthesis. The use of Shilajit likely contributed to the formation of stable, well‐defined NPs due to its rich organic matrix containing fulvic acid and other reducing agents.

FIGURE 6TEM images of (a) NiO NPs and (b) alumina NPs synthesized using Shilajit extract.

**Figure:**
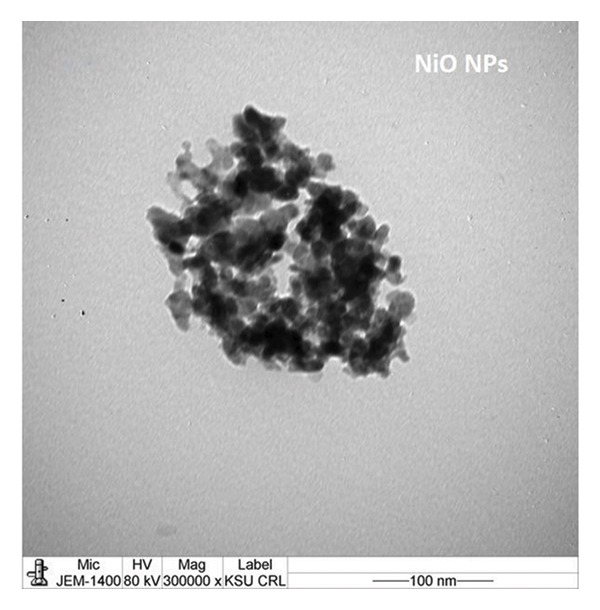
(a)

**Figure:**
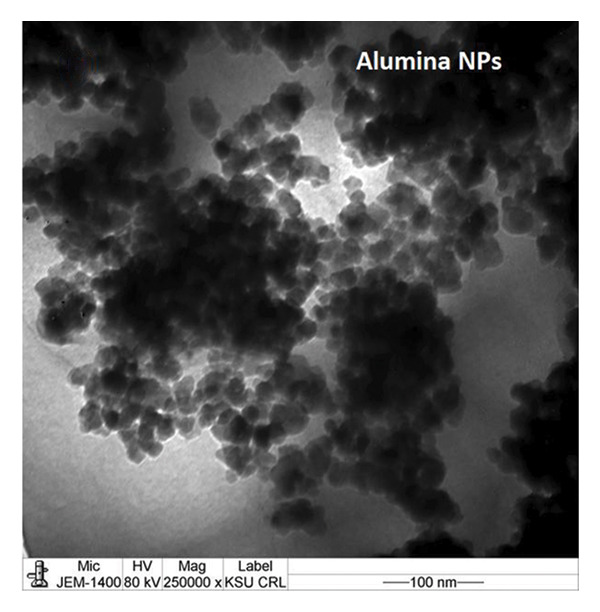
(b)

SEM coupled with EDX was used to investigate the elemental composition of alumina and NiO NPs synthesized with Shilajit extract. This integrated approach provided detailed insights into both the morphology and elemental distribution of the NPs. EDX analysis provided information on the weight percentage composition of the NPs and revealed the dominance of certain elements. For the alumina NPs, the weight percentage was 75.88% oxygen (O) and 24.12% aluminum (Al), confirming a dominant presence of oxygen with a significant amount of aluminum. For the NiO NPs, the weight composition was 42.2% oxygen (O) and 57.8% nickel (Ni), indicating a balanced ratio of nickel and oxygen, with both elements equally represented in the NP structure. These results highlight the remarkable dominance of aluminum and nickel in the synthesized NPs and emphasize their essential role in the formation of materials (Figures 7(a) and 7(b)).

FIGURE 7Elemental composition of (a) alumina NPs and (b) NiO NPs synthesized using Shilajit extract.

**Figure:**
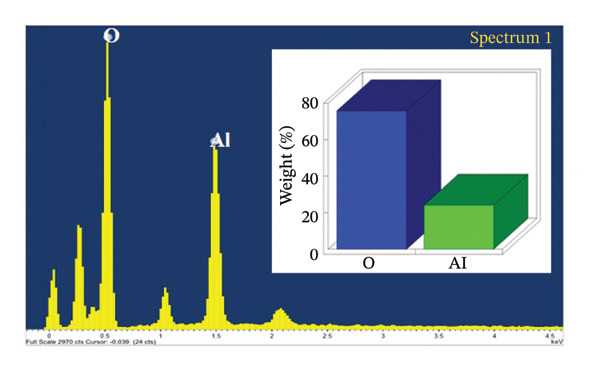
(a)

**Figure:**
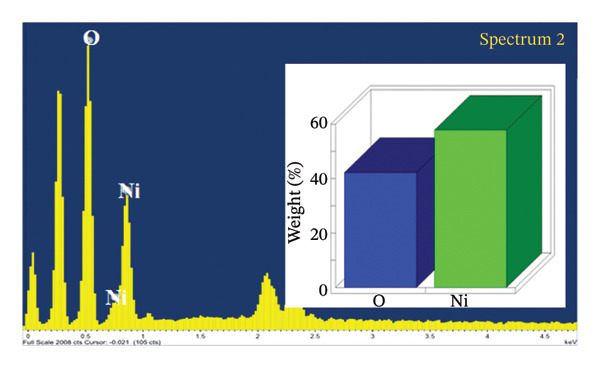
(b)

Elemental mapping is essential for confirming the composition, distribution, and uniformity of NPs. It enables accurate identification of the elements present, such as Al, Ni, and O in alumina NPs and NiO NPs, and ensures proper stoichiometry and synthesis. The mapping also helps to verify the homogeneity of the distribution of the NPs, confirms their size and shape, and provides information on surface properties, such as coating or functionalization. Elemental mapping is essential to confirm that the NPs meet the intended specifications and to identify any impurities or structural inconsistencies. The combined information from SEM, EDX, and elemental mapping contributes significantly to verifying the effective synthesis and detailed characterization of alumina and NiO NPs, as shown in Figures 8(a), 8(b), 8(c), 8(d), 8(e), 8(f).

FIGURE 8Mapping images of (a–c) NiO NPs and (d–f) alumina NPs.

**Figure:**
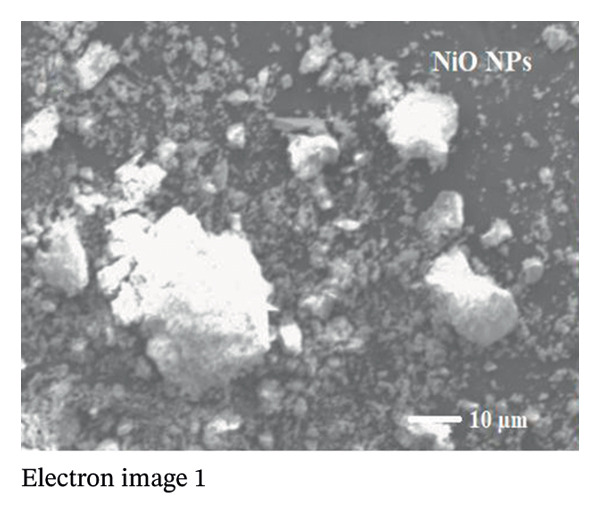
(a)

**Figure:**
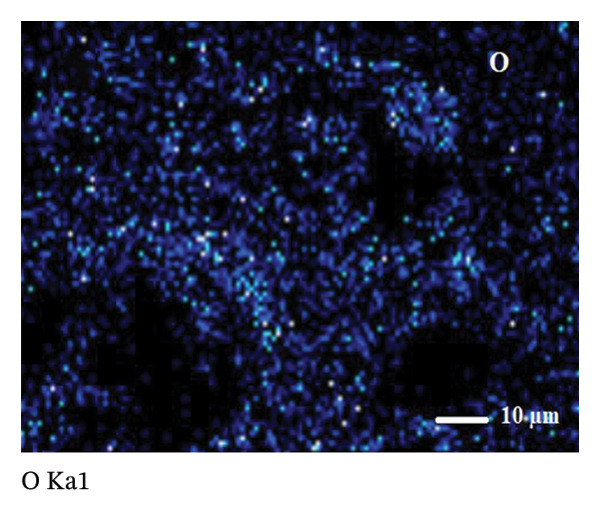
(b)

**Figure:**
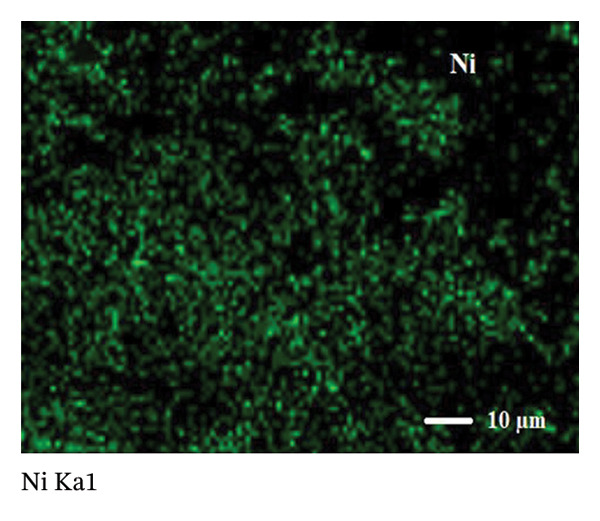
(c)

**Figure:**
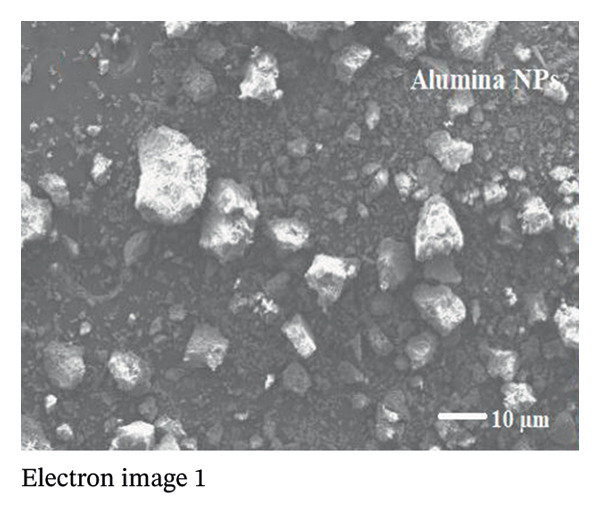
(d)

**Figure:**
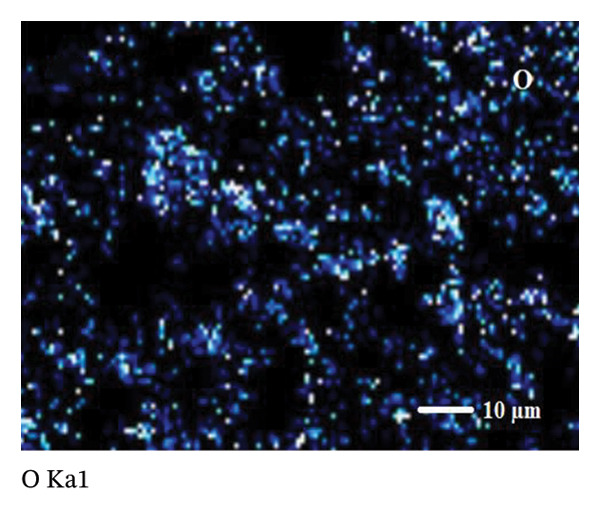
(e)

**Figure:**
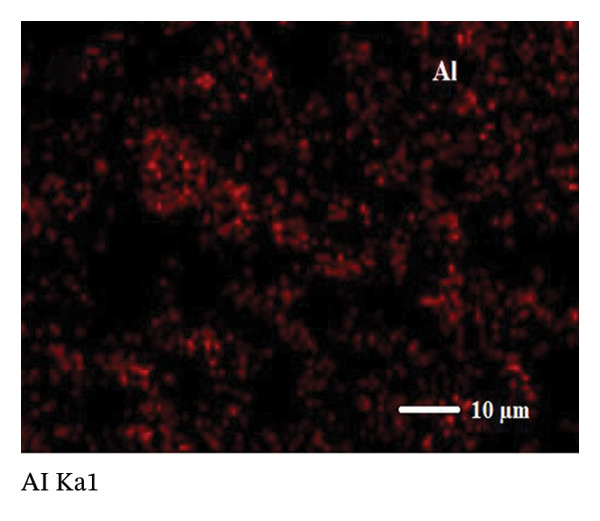
(f)

### 3.2. Spectral Features

FL analysis of PRM in the presence of SDS/alumina NPs and SDS/NiO NPs investigates the interactions between these components and their combined influence on detection sensitivity and selectivity. PRM shows energy absorption at an excitation wavelength of 350 nm and emits fluorescence at 400 nm, which corresponds to its characteristic emission peak (Figures 9(a) and 9(b)). However, the presence of SDS, an anionic surfactant, plays a crucial role in improving the solubility of PRM in aqueous solutions, allowing it to interact more effectively with the NPs. The SDS molecule facilitates the formation of micelles, which improves the dispersibility of PRM and the NPs in the solution, thus increasing the surface area for interaction. Alumina and NiO NPs contribute to this process by influencing the photophysical properties of PRM. These metal oxide NPs act as fluorescence enhancers by creating a unique microenvironment around PRM that can alter its electronic states. This change in electronic states leads to an increase in fluorescence quantum yield, which ultimately improves the sensitivity of fluorescence detection.

FIGURE 9Fluorescence spectra of 5.0 μg/mL of (a) piracetam, SDS, piracetam‐SDS, alumina NPs, piracetam‐alumina NPs, and piracetam‐SDS‐alumina NPs and (b) piracetam, SDS, piracetam‐SDS, NiO NPs, piracetam‐ NiO NPs, and piracetam‐SDS‐NiO NPs.

**Figure:**
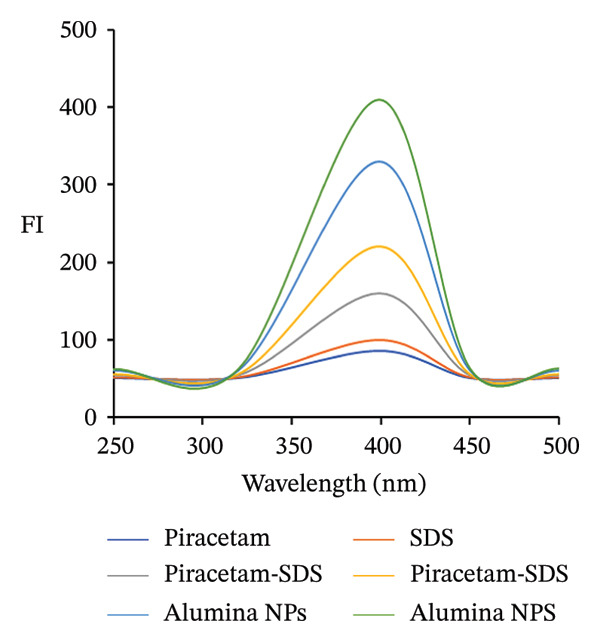
(a)

**Figure:**
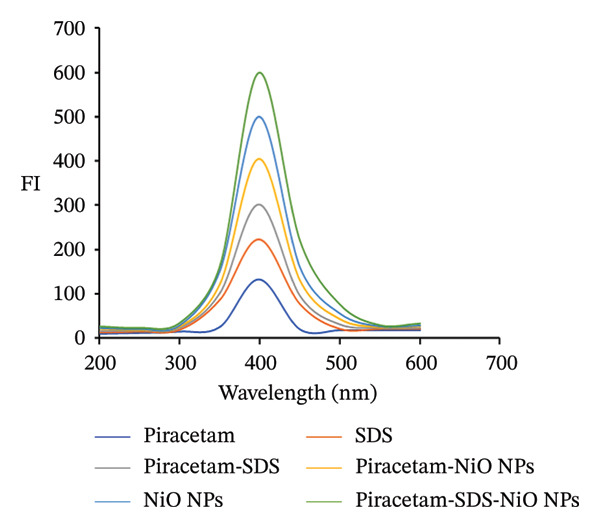
(b)

In addition, these NPs also serve as light scattering agents that can enhance the fluorescence signal by preventing photobleaching or increasing the efficiency of photon absorption and emission. The combined effect of the surfactant, the metal oxide NPs, and the modified electronic environment significantly improves the overall FI of PRM and enables more accurate and sensitive detection in the assay study.

### 3.3. Refinement of Experimental Conditions

The fluorescence detection method for PRM has been thoroughly refined by systematically varying critical parameters to improve both sensitivity and precision. One of the most critical factors is the choice of solvent, which directly affects the solubility of PRM and its fluorescence behavior, and ensures that the analyte is in an optimal state for detection. The optimization of surfactants, especially SDS, plays a crucial role as it increases the interaction between PRM and the fluorescence‐enhancing agents, including metal oxide NPs, allowing for more efficient energy transfer.

The concentration and volume of alumina NPs and NiO NPs are also fine‐tuned, as their presence significantly increases FL intensity through effective energy transfer mechanisms between the NPs and the analyte. Buffer solutions, including their type and concentration, are another important factor as they maintain the stability of the PRM solution and ensure that the pH is in a range that supports the optimal ionization state of the drug, which in turn influences the fluorescence properties. In addition, the response time was carefully evaluated to determine the most appropriate time for the fluorescence measurements and to ensure that the signal was captured at its peak. By precisely tuning all these parameters, which are summarized in Table 1, the method results in a robust and reproducible system for the accurate detection of PRM and provides improved sensitivity and reliability for analytical purposes.

**TABLE 1:** Adjustment of analytical conditions for determination of PRM using SDS/alumina NPs and SDS/NiO NP fluorescence systems.

Analytical parameters	Experimental range	Alumina NPs/NiO NPs
*λ*_ex_/*λ*_em_ wavelength (nm)	200–900	350/400
Type of buffer solution	Acetate, phosphate, and borate	Acetate
pH of the buffer	3–10	4
Volume of buffer (mL)	0.5–3.0	2.0
Volume of NPs	0.5–3.0	2.0/1.5
Type of surfactant	SDS, Triton X‐100, CPC	SDS
Volume of surfactant (mL)	0.1–3.0	0.2
Time of determination (min)	1–10	3

### 3.4. Effect of Solvent

The choice of solvent is of crucial importance for the FI of a substance, as it influences the solution environment and thus the electronic properties of the fluorescent molecules. Various solvents, including water, ethanol, methanol, and acetonitrile, were investigated for their influence on the FI of a 5.0 μg/mL PRM solution. The FI of a compound can be significantly affected by the choice of solvent, as the solvent influences both the electronic environment of the molecule and the dynamics of its excited states. In the case of water, ethanol, methanol, and acetonitrile, each solvent has different properties that change the fluorescence behavior. Water, with an FI of 150, is strongly polar and can effectively solve the excited states of the fluorescent molecule, resulting in relatively strong emission intensities.

However, the strong polarity of water can also cause some degree of quenching by enhanced nonradiative relaxation, especially if the solvent stabilizes certain excited states that facilitate energy loss without emission. Ethanol with FI 160, which is less polar than water, can lead to weaker solvation of excited states, which can either enhance fluorescence due to reduced quenching or reduce it if the interaction with the solute is insufficient to stabilize the excited state. Methanol with FI 152 reduces polarity even further compared to ethanol and water, which could provide a more favorable environment for fluorescence in certain cases, especially with less polar fluorophores, as it tends to minimize nonradiative paths and promote radiative transitions.

**FIGURE 10:**
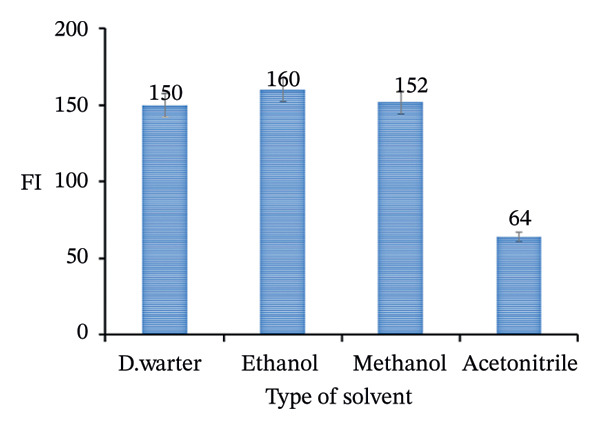
Effect of different solvents on the FI of 5.0 μg/mL PRM.

Acetonitrile with FI 64, which is less polar than all other solvents, gives the lowest FI in this series. The lower polarity and lower solvation capacity of acetonitrile often lead to a weaker interaction with the excited states of the fluorescent species, which can lead to a significant increase in nonradiative relaxation processes, and thus to a lower emission intensity. The choice of deionized water as a solvent in this analysis is particularly advantageous for minimizing toxicity issues, as water is a nontoxic, environmentally friendly solvent, that is consistent with green chemistry principles (Figure 10).

### 3.5. Effect of the Buffer Solution

Various pH values between 3 and 10 were tested with different buffer solutions, including acetate, phosphate, and borate, to determine the most favorable environment for PRM fluorescence. In this study, the selection of an acetate buffer with a pH of 4 was critical for optimizing the FI in the determination of PRM. The acetate buffer was found to be the most effective as it provides a stable and uniform ionic strength, which is essential for maintaining the PRM compound in solution without aggregation or precipitation. The specific pH of 4 is particularly suitable for PRM as it likely corresponds to the optimal fluorescence conditions of the molecule and ensures that the compound remains in its most stable and fluorescent state (Figure 11(a)).

FIGURE 11Adjustment of different analytical parameters that affect the FI of PRM: (a) type of buffer, (b) volume of acetate buffer, (c) type of surfactants, and (d) volume of SDS.

**Figure:**
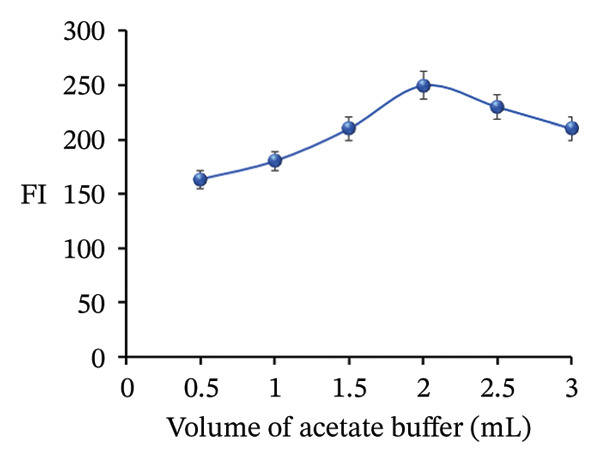
(a)

**Figure:**
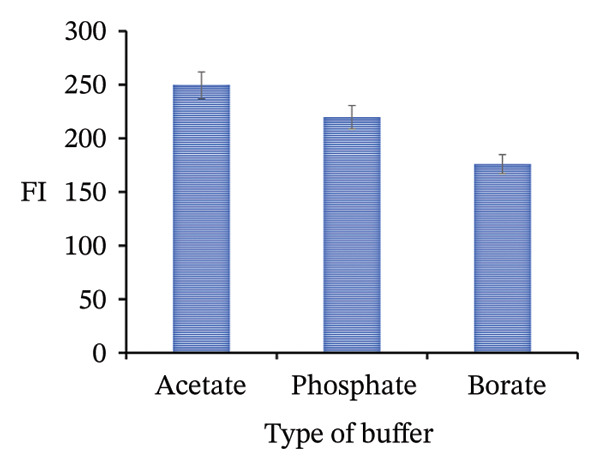
(b)

**Figure:**
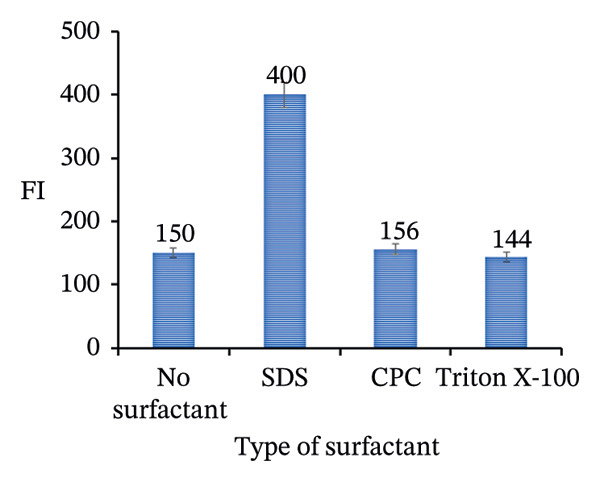
(c)

**Figure:**
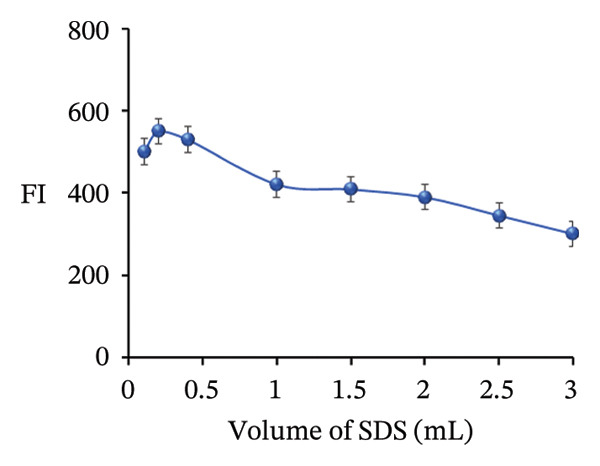
(d)

The volume of pH 4 acetate buffer was tested in the range of 0.5–3 mL to determine the optimal volume for enhancing FI during PRM determination. It was found that the ideal volume to achieve the highest FI was 2.0 mL. This volume likely provided the right balance between ionic strength and solvation capacity and ensured that the PRM remained well dissolved and stable in the solution while creating an optimal environment for FI. A smaller volume (e.g., 0.5 mL) may not have provided sufficient buffering capacity to maintain a stable ionic environment, while larger volumes (e.g., 3.0 mL) may have diluted the solution too much, resulting in lower PRM concentrations and consequently lower fluorescence. Therefore, 2.0 mL was determined to be the most effective volume to maximize FI in this experiment (Figure 11(b)).

### 3.6. Effect of Type and Volume of Surfactant

The influence of three different surfactants 0.01 mol/L SDS, Triton X‐100, and CPC on the FI of PRM was investigated. The results showed that SDS significantly increased the FI even in the absence of NPs, highlighting its effective role in enhancing PRM fluorescence. In contrast, both the cationic surfactant CPC and the nonionic surfactant Triton X‐100 were found to decrease FI, as shown in Figure 11(c). To further optimize the process, the ideal SDS volume was determined, which varied between 0.1 and 3.0 mL. As shown in Figure 11(d), the optimal volume was 0.2 mL, which resulted in the highest peak of FI. These results emphasize the crucial role of selecting the appropriate surfactant and concentration to improve the fluorescence detection of PRM.

### 3.7. Effect of NP Volume

To identify the ideal concentrations of alumina NPs and NiO NPs for the fluorescence system, a range of test samples was prepared with varying NP volumes, from 0.5 to 3.0 mL. The FI was recorded for each sample, revealing a distinct peak at 2.0 mL for alumina NPs and 1.5 mL for NiO NPs. These volumes resulted in the strongest emission signal, indicating that they strike an optimal balance between enhancing fluorescence and minimizing the potential quenching effects seen at higher concentrations. Consequently, 2.0 mL of alumina NPs and 1.5 mL of NiO NPs were chosen as the optimal volumes for subsequent experiments (Figure 12(a)).

FIGURE 12(a) Effect of nanoparticle volume, (b) effect of temperature, and (c) effect of response time on the PRM‐SDS‐alumina NPs and PRM‐SDS‐NiO NPs fluorescence systems.

**Figure:**
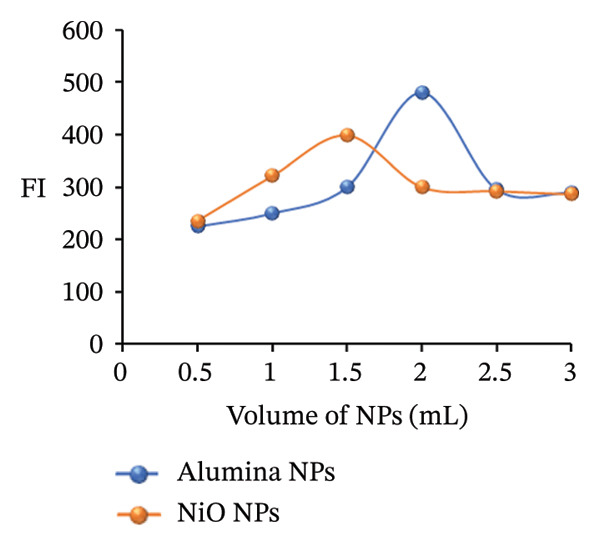
(a)

**Figure:**
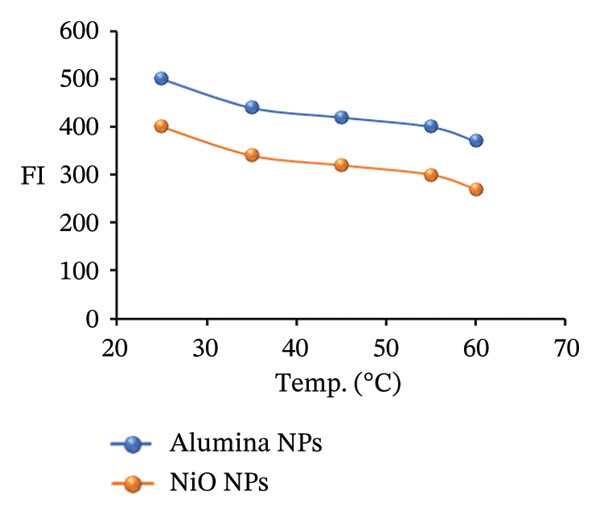
(b)

**Figure:**
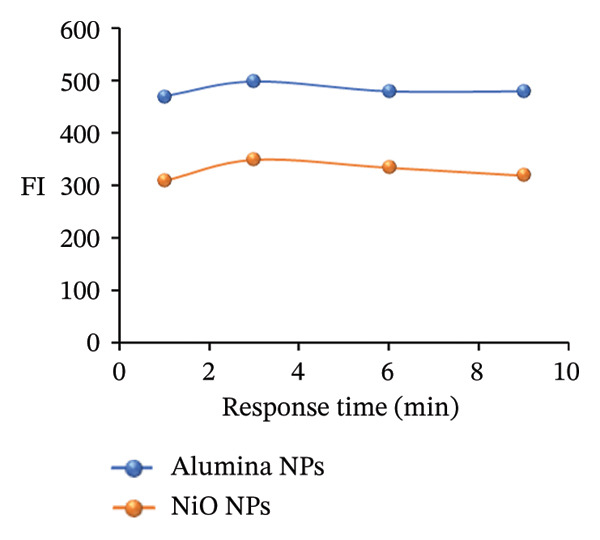
(c)

### 3.8. Effect of Temperature

The FI of a PRM standard solution with a constant concentration of 1.0 μg/mL was investigated over a temperature range of 25°C–60°C (Figure 12(b)). The aim was to assess how different thermal conditions affect the fluorescence behavior of the solution. The FI was measured at each specific temperature point, to observe any temperature‐dependent changes. The results clearly showed that the FI progressively decreased with increasing temperature. This decrease in FI at increasing temperatures can be attributed to the increased molecular motion at higher temperatures. In addition, changes in solvent properties, such as a reduction in viscosity or variations in dielectric constant, can alter solvation dynamics and reduce the overall quantum yield of fluorescence. Another important factor is that elevated temperatures can cause the fluorophores to become thermally unstable, leading to structural degradation or even thermal breakdown, which further attenuates the fluorescence signal.

### 3.9. Effect of Response Time

To investigate how reaction time affects the FI of a PRM standard, mixtures were prepared by adding 2.0 mL alumina NPs or 1.5 mL NiO NPs to 0.2 mL SDS. The mixtures were monitored at intervals of 1–10 min. Measurements taken throughout the course showed that FI increased gradually, with a marked increase after the third minute. This result suggests that the interaction between PRM, alumina or NiO NPs, and SDS reaches optimal fluorescence after this time interval, indicating a fast and efficient reaction. As shown in Figure 12(c), these results emphasize the critical role of reaction time in improving the FL properties of the PRM solution, which could affect its detection and quantification performance.

### 3.10. Method Validation

The proposed spectrofluorimetric technique has been extensively validated following the standards established by the International Council for Harmonization (ICH) [37]. To determine linearity for PRM, FIs were recorded and plotted against concentrations between 0.5 and 10 μg/mL for the PRM‐SDS‐alumina NP system and 0.2 and 14 μg/mL for the PRM‐SDS‐NiO NP system. The resulting regression equations, FI = 97.393*x* − 21.748 and FI = 49.658*x* + 29.776 for both systems, yielded impressive correlation coefficients (*r* ^2^) of 0.9997 and 0.9998, respectively, indicating a strong linear relationship across the concentration ranges studied. This excellent linearity underlines the reliability and effectiveness of the method for the accurate quantification of PRM in different sample types (Figures 13(a), 13(b), 13(c), 13(d)).

FIGURE 13(a, c) Linear regression graphs and (b, d) FI of various concentrations of PRM solutions using PRM‐SDS‐alumina NP and PRM‐SDS‐NiO NP fluorescence systems, respectively.

**Figure:**
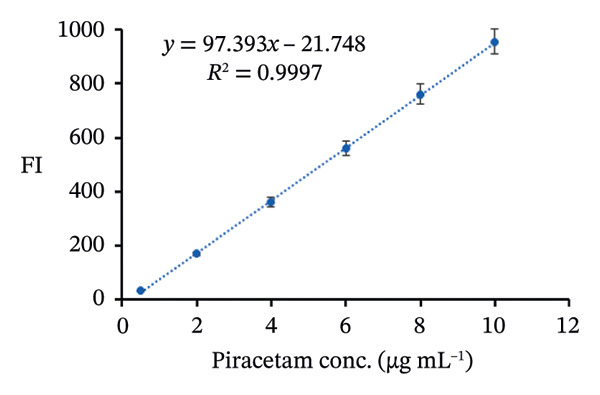
(a)

**Figure:**
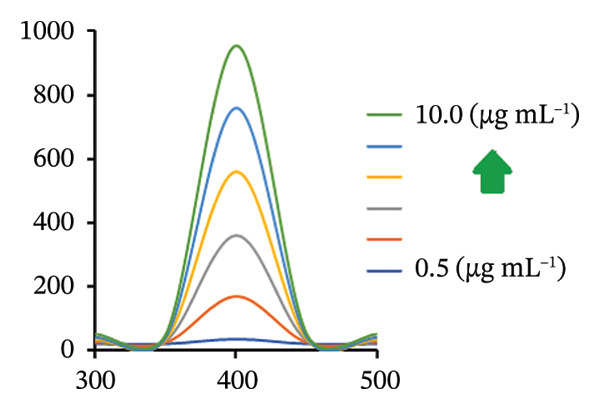
(b)

**Figure:**
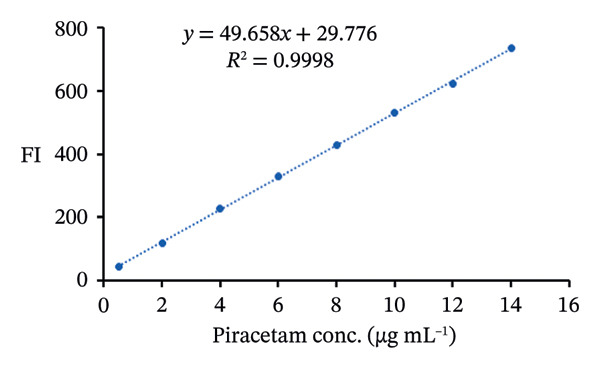
(c)

**Figure:**
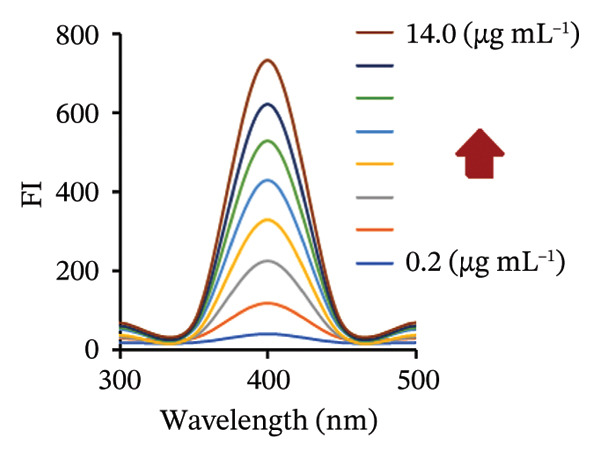
(d)

Statistical analysis of the data revealed an extraordinarily high correlation coefficient of *r* = 0.9999, indicating a perfect correlation between the concentrations of FI and PRM. This strong correlation proves the precision of the method for quantitative measurements. The robust linearity of the method is further emphasized by the low standard deviations for the slope (Sb = 0.7753, Sb = 29.776) and intercept (Sa = 4.6976, Sa = 0.9422). These small deviations show that the regression line fluctuates very little, indicating that the fluorescence response is stable and predictable over the concentration range studied. Table 2 contains a detailed summary of these results.

**TABLE 2:** Analytical data obtained from the spectrofluorimetric determination of PRM using PRM‐SDS‐alumina NP and PRM‐SDS‐NiO NP fluorescence systems.

Parameters	PRM‐SDS‐alumina NPs	PRM‐SDS‐NiO NPs
Regression equation	FI = 97.393*x* − 21.748	FI = 49.658*x* + 29.776
Correlation coefficient (*r* ^2^)	0.9997	0.9998
Slope	97.393	49.658*x*
Intercept	21.748	29.776
Sb	0.7753	0.1126
Sa	4.6976	0.9422
Concentration range (μg/mL)	0.5–10	0.2–14
Detection limit	0.159	0.063
Quantification limit	0.482	0.189

The lower limits of detection (LOD) and quantification were calculated using Equations (6) and (7) as described in [38].

(6) 3.3×σblankS,

(7) 10×σblankS.

The analysis of PRM using the PRM‐SDS‐alumina NP and PRM‐SDS‐NiO NP fluorescence systems gave excellent results, as shown in Table 2. This method showed exceptional sensitivity, with LOD of 0.159 and 0.063 μg/mL and limits of quantification (LOQ) of 0.482 and 0.189 μg/mL for the respective systems. The precision of nine concentrations of PRM (0.5–10 μg/mL) for the system modified with alumina and (0.2–14 μg/mL) for the system modified with NiO NPs resulted in an average recovery of 98.79% ± 1.07% and 99.13% ± 0.64%, respectively. The estimated percent standard error (%SE) and relative standard deviation (%RSD) of 0.36%, 0.21%, 1.08%, and 0.64% for the PRM‐SDS‐alumina NP and PRM‐SDS‐NiO NP fluorescent systems, respectively, demonstrate the consistency of the method, which is summarized in Table 3.

**TABLE 3:** Accuracy data obtained from the spectrofluorimetric determination of PRM using PRM‐SDS‐alumina NP and PRM‐SDS‐NiO NP fluorescence systems.

	**PRM-SDS-alumina NPs**			**PRM-SDS-NiO NPs**		
	**Taken (ppm)**	**Found (ppm)**	**% recovery**	**Taken (ppm)**	**Found (ppm)**	**% recovery**
Statistical analysis	0.5	0.49	98.00	0.2	0.2	100.00
	2.0	1.98	99.00	0.5	0.49	98.00
	2.5	2.51	100.70	2.0	1.99	99.50
	4.0	3.94	98.50	4.0	3.96	99.00
	4.5	4.50	100.00	6.0	5.89	98.17
	6.0	5.83	97.20	8.0	7.93	99.13
	6.5	6.42	98.90	10.0	9.97	99.70
	8.0	7.94	99.30	12.0	11.95	99.58
	10.0	9.75	97.50	14.0	13.87	99.07

Mean ± SD	98.79 ± 1.07			99.13 ± 0.64		
*n*	9			9		
Variance	1.14			0.40		
%SE	0.36			0.21		
%RSD	1.08			0.64		

Precision by intra‐ and interday assays at doses of 0.5, 6.0, and 10 μg/mL for the modified alumina NP system and 0.2, 8.0, and 14.0 μg/mL for the modified NiO NP system, evaluated three times each, yielded intra‐ and interday %RSDs of 0.11%, 0.40%, 1.72% and 1.45%, 1.34%, 0.02% for PRM‐SDS‐alumina NPs, respectively. In contrast, the values within one day (0.09%, 0.02%, and 0.01%) and between 2 days (0.04%, 0.06%, and 0.02%) were determined for the PRM‐SDS‐NiO NP fluorescence system. The %RSD of both systems was below 2%, indicating high precision (Table 4).

**TABLE 4:** Precision data obtained from the spectrofluorimetric determination of PRM using PRM‐SDS‐alumina NP and PRM‐SDS‐NiO NP fluorescence systems.

Precision		Taken (ppm)	% recovery ± SD	% RSD	% error
Piracetam‐SDS‐alumina NPs	Intraday assay	0.5	97.89 ± 0.1	0.11	0.06
		6.0	99.76 ± 0.4	0.40	0.23
		10.0	99.00 ± 1.7	1.72	0.98
	Interday assay	0.5	99.17 ± 1.44	1.45	0.83
		6.0	99.23 ± 1.33	1.34	0.77
		10.0	98.44 ± 0.02	0.02	0.01

Piracetam‐SDS‐NiO NPs	Intraday assay	0.2	99.91 ± 0.09	0.09	0.05
		8.0	99.96 ± 0.02	0.02	0.01
		14.0	99.94 ± 0.01	0.01	0.01
	Interday assay	0.2	99.92 ± 0.04	0.04	0.02
		8.0	99.90 ± 0.06	0.06	0.03
		14.0	99.97 ± 0.02	0.02	0.01

The stability of the PRM‐SDS‐alumina NP and PRM‐SDS‐NiO NP fluorescence systems was thoroughly tested by subjecting them to minor changes in key process parameters. The pH of the solution was adjusted by ±1 unit, the surfactant concentration by ±0.1, and the volumes of alumina NPs and NiO NPs by ±0.2 mL. These changes were made to simulate possible variations in the experimental conditions. The results showed that these variations had no significant effect on the recovery rates, which remained constant at 98.50 ± 1.17 for the PRM‐SDS‐alumina NP system and 98.97 ± 0.70 for the PRM‐SDS‐NiO NP system. This constant recovery rate demonstrates the reliability and robust performance of the fluorescence systems, even with small process variations. This stability is essential to ensure reliable and reproducible results in different laboratory environments and underlines the suitability of the method for routine PRM analysis.

The effect of various interfering factors, such as amino acids (glycine, L‐tyrosine, L‐histidine, and L‐threonine), sugar (lactose), cations (Fe^3+^, Cu^2+^, Na^+^, K^+^, Mg^2+^, Zn^2+^, and Ca^2+^), and additives (such as magnesium stearate, titanium dioxide, colloidal anhydrous silicon dioxide, and hydroxypropylmethylcellulose) on the selectivity of the PRM‐SDS‐alumina NP and PRM‐SDS‐NiO NP fluorescence systems. The interferents were introduced at a concentration of 0.1 μg/mL, while the PRM samples were maintained at 5.0 μg/mL. The data, summarized in Table 4, illustrate the effectiveness of the system in discriminating between PRM and potential interferents. Equation (8) was used to calculate the acceptable deviation of the fluorescence signal.

(8) Tolerable fluorescence deviation=Imeasured−IexpectedIexpected.

In this analysis, *I*_measured_ and *I*_expected_ are the FIs of the analyte in the presence of interferences and FI of the analyte without interferences. To keep the interference within tolerable limits, the deviation should stay within an acceptable percentage, often set as a threshold (e.g., ±5). Table 5 shows the tolerable values for the assay of PRM using PRM‐SDS‐alumina NP and PRM‐SDS‐NiO NP fluorescence systems.

**TABLE 5:** Selectivity coefficient for the assay of PRM using PRM‐SDS‐Al_2_O_3_ NP and PRM‐SDS‐NiO NP fluorescence systems.

Interferences	Tolerable value	Tolerable value
	PRM‐SDS‐alumina NPs	PRM‐SDS‐NiO NPs
Lactose	362	193
Glycine	193	219
L‐Tyrosine	201	205
L‐Histidine	222	197
L‐Threonine	233	165
Fe^3+^	203	145
Cu^2+^	520	600
Na^+^	188	375
K^+^	612	702
Mg^2+^	645	841
Zn^2+^	301	514
Ca^2+^	184	137
Magnesium stearate	320	403
Titanium dioxide	290	325
Colloidal anhydrous silica	265	312
Hydroxypropyl methylcellulose	240	285

### 3.11. Analytical Applications

With recoveries of 99.07% ± 0.65% and 99.60% ± 0.37%, respectively, the fluorescence methods using PRM‐SDS‐alumina NPs and PRM‐SDS‐NiO NPs to measure PRM concentrations in pure powder showed remarkable accuracy. These results demonstrate the exceptional sensitivity of the fluorescence systems, which is due to the special properties of the alumina and NiO NPs. Both materials have a large surface area, which improves their interaction with PRM molecules, resulting in more efficient binding and better detection performance. In addition, their mechanical strength ensures long lifetime during analysis, while their large band gaps contribute to superior fluorescence properties, resulting in high sensitivity and precision of measurement. The combination of these factors enables the proposed systems to achieve exceptional performance in the detection of PRM concentrations, which is also confirmed by the data presented in Table 6.

**TABLE 6:** Spectrofluorimetric determination of piracetam in bulk powder using PRM‐SDS‐alumina NP and PRM‐SDS‐NiO NP fluorescence systems.

	**PRM-SDS-alumina NPs**			**PRM-SDS-NiO NPs**		
	**Taken (ppm)**	**Found (ppm)**	**%recovery**	**Taken (ppm)**	**Found (ppm)**	**%recovery**
Statistical analysis	0.5	0.49	98.0	0.2	0.199	99.5
	2.0	1.97	98.5	2.0	1.98	99.0
	4.0	3.96	99.0	4.0	3.97	99.3
	6.0	5.97	99.5	8.0	8.00	100.0
	8.0	7.96	99.5	10.0	9.94	99.4
	10.0	9.99	99.9	12.0	12.00	100.0
				14.0	14.00	100.0

Mean ± SD	99.07 ± 0.65			99.60 ± 0.37		
*n*	6			6		
Variance	0.42			0.14		
%SE	0.27			0.14		
%RSD	0.65			0.37		

In addition, the fluorescence methods PRM‐SDS‐alumina NPs and PRM‐SDS‐NiO NPs were used to determine the PRM concentration in tablet dosage forms. The results were 99.49% ± 0.45% and 99.54% ± 0.46%, respectively, demonstrating the high accuracy of the methods in real applications. To evaluate the validity of these results, a statistical analysis was performed using Student′s *t*‐test and the variance ratio F‐test. The results of this analysis confirmed that the investigated FL systems are reliable for the quantification of PRM in commercial samples. This statistical evaluation played a crucial role in comparing the new methods with an established, well‐known analytical technique.

The established method uses a simple, environmentally friendly, and cost‐effective spectrofluorimetric approach that makes use of the sensitizing effect of the cationic surfactant SDS. After excitation at 350 nm, the method produces a maximum FI at 400 nm. The comparison of these results shows that the PRM‐SDS‐alumina NP and PRM‐SDS‐NiO NP fluorescence methods not only provide comparable results but also represent a reliable alternative for precise PRM determination. This comparison underlines the role of nanotechnology in the further development of analytical techniques that offer improved sensitivity and efficiency in the detection of target compounds, such as PRM (Table 7).

**TABLE 7:** Spectrofluorimetric determination of PRM tablet (piracetam Al® 800 mg/tablet) using PRM‐SDS‐alumina NP and PRM‐SDS‐NiO NP fluorescence systems.

	**PRM-SDS-alumina NPs**			**PRM-SDS-NiO NPs**		
	**Taken (ppm)**	**Found (ppm)**	**%recovery**	**Taken (ppm)**	**Found (ppm)**	**%recovery**
Statistical analysis	0.5	0.50	100.00	0.2	0.2	100.00
	2.0	1.98	99.00	2.0	1.99	99.50
	4.0	4.00	100.00	4.0	3.99	99.75
	6.0	5.96	99.33	8.0	7.96	99.50
	8.0	7.92	99.00	10	9.86	98.60
	10.0	9.96	99.60	14	13.98	99.86

Mean ± SD	99.49 ± 0.45			99.54 ± 0.46		
*n*	6			6		
Variance	0.21			0.20		
%SE	0.18			0.16		
%RSD	0.45			0.46		
*t*‐test	0.431 (2.228)^∗^			0.520 (2.228)^∗^		
F‐test	1.71 (5.05)^∗^			1.80 (5.05)^∗^		

^∗^Tabulated Student’s *t*‐test and F‐test at *p* = 0.05.

### 3.12. Possible Mechanisms of Fluorescence Systems in the Determination of PRM

The mechanism underlying the interaction between PRM and the Shilajit‐derived metal oxide nanosensors primarily involves surface adsorption, hydrogen bonding, and charge transfer interactions. The nanosensors, synthesized from Shilajit, a complex natural organic matrix rich in fulvic acid, humic substances, and trace metals, are thermally treated to produce metal oxide NPs with high surface area and active surface functional groups (e.g., hydroxyl and carboxyl). These functional groups on the NP surface enable strong intermolecular interactions with PRM through hydrogen bonding between the drug’s amide and carbonyl groups and the surface –OH or –COOH groups. Additionally, π–π stacking and dipole–dipole interactions may occur due to the presence of aromatic and polar structures in both the drug and the organic remnants from Shilajit. Upon interaction, these molecular‐level contacts alter the electron density and surface charge of the nanosensor, modulating its fluorescence properties by enhancing emission. This fluorescence response is key to detection and quantification. Moreover, the study reveals how natural matrices, such as Shilajit, can be engineered through calcination and doping to form nanostructured oxides with tunable surface chemistry and electronic properties, effectively converting a raw organic substance into a functional, sensitive, and environmentally benign sensing platform. Thus, the possible mechanism behind the PRM‐SDS‐alumina NP and PRM‐SDS‐NiO NP fluorescent systems for the determination of PRM involves a combination of physical interactions and fluorescence properties that are enhanced by the NPs and surfactant. The process begins with the interaction of PRM with the cationic surfactant, SDS, which acts as a stabilizer for the NPs (alumina and NiO) and facilitates their dispersion in the solution. The SDS molecules form micelles or aggregates that can bind to PRM molecules through electrostatic interactions or hydrophobic effects, depending on the type of PRM. This binding significantly increases the sensitivity and selectivity of the system.

Once PRM is in the presence of SDS and the NPs, the FL process is activated. The alumina and the NiO NPs play a crucial role in this process due to their unique surface properties. The large surface area of these NPs enables a strong interaction with PRM molecules, which leads to the formation of a stable complex. In addition to physical binding, the wide band gap and mechanical robustness of the NPs contribute to their ability to efficiently absorb the excitation energy and release it as FL.

Upon excitation with a specific wavelength (in this case 350 nm), the NPs (especially NiO, which has significant absorption in the UV spectrum) absorb the energy and transfer it to the PRM or the SDS‐bound complex. This energy transfer facilitates the emission of light, resulting in a measurable FL signal, usually observed at 400 nm. The FI is closely related to the PRM concentration, as a higher PRM concentration leads to a stronger interaction with the NPs, and thus increases the FL output. In addition, the special characteristics of alumina and NiO NPs, especially their wide band gap (which prevents unnecessary energy loss through nonradiative processes), their excellent optical properties, and their ability to stabilize the PRM‐SDS complex, contribute to their ability to promote FL emission very efficiently.

The inclusion of SDS further optimizes the system by increasing the solubility of the NPs in aqueous solutions and improving their interaction with PRM molecules. This surfactant not only helps to stabilize the NPs but also increases the total surface area available for PRM binding. In addition, the combination of SDS with the NPs prevents agglomeration and maintains the ideal size and surface properties of the NPs, further increasing the sensitivity of the system.

PRM‐SDS‐alumina NP and PRM‐SDS‐NiO NP FL systems for the determination of PRM were compared with the results of previous studies using different analytical techniques. An RP‐HPLC technique was used for the determination of PRM. Arayne et al. [39] investigated the simultaneous detection of PRM and its four impurities using triethylamine: acetonitrile (85: 15 v/v), the linearity was 50–55 ng/mL and the LOD was 25 ng/mL. Another RP‐HPLC method was described by Siddiqui et al. [40]. They investigated the determination of PRM in pharmaceuticals and biological fluids using a mixture of 0.1 g/L triethylamine: acetonitrile (70: 30 v/v). The results covered the concentration range of 20–1000 ng/mL and LOD of 2.43 ng/mL. Furthermore, Mansour et al. [5] also used mobile phase (methanol, acetonitrile, dis. H_2_O, 10:20:70 v/v/v) for the RP‐HPLC determination of PRM over a concentration range of 10–100 μg/mL and LOQ of 2.7 μg/mL. The chromatographic method coupled with mass spectrometry was developed by Wang et al. [41] using trichloroacetic acid (5%). Detection was performed over the concentration range of 0.1–20 μg/mL and LOQ of 0.1 μg/mL. Two spectrophotometric methods were described by Livia et al. [42] and Dhoru et al. [43] using a methanolic solution of 0.1 M HCl and distilled water, respectively.

The measurements were carried out using concentration ranges of 5–40 and 4.0–28 μg/mL and LOD of 0.369 and 0.42 μg/mL, respectively. A spectrofluorimetric method was described by Omar et al. [10]; in this study, the reagent used was eosin Y and the determination was made using a concentration range of 0.3–3.0 μg/mL and LOD of 0.09 μg/mL. The proposed fluorescence systems stand out due to their exceptional environmental sustainability, low detection thresholds, ease of use, and affordability. In comparison with chromatographic and spectroscopic techniques, these systems provide higher precision and accuracy while demanding less specialized knowledge, as shown in Table 8. This makes the PRM‐SDS‐alumina NP and PRM‐SDS‐NiO NP fluorescence methods highly effective alternatives for PRM detection.

**TABLE 8:** A comparison of the analytical results from previously established methods with the current spectrofluorimetric assays involving PRM‐SDS‐alumina NPs and PRM‐SDS‐NiO NPs.

Method of analysis	Reagents	Linear range	LOD/LOQ	Ref.
PR‐HPLC	Triethylamine: acetonitrile (85: 15 v/v)	50–55 ng/mL	25 ng/mL	[39]
RP‐HPLC/UV detection	Mixture of 0.1 g/L of triethylamine: acetonitrile (70 : 30 v/v)	20–10000 ng/mL	2.43 ng/mL	[40]
RP‐HPLC	(methanol, acetonitrile, dis.H_2_O) (10:20:70 v/v/v)	10–100 ppm	2.7 LOQ ppm	[5]
LC‐MS‐MS	Trichloroacetic acid (5%)	0.1–20 ppm	0.1 ppm (LOQ)	[41]
Spectrophotometry	0.1 M HCl methanolic solution	5–40 ppm	0.369 ppm	[42]
Spectrophotometry	Distilled water	4.0–28 ppm	0.42 ppm	[43]
Spectrofluorimetry	Eosin Y	0.3–3.0 ppm	0.09 ppm	[10]
Current work	PRM‐SDS‐alumina NPs PRM‐SDS‐NiO NPs	0.5–10.0 ppm 0.2–14.0 ppm	0.159 ppm 0.063 ppm	

### 3.13. Environmental Impact Assessments of the Developed Systems

#### 3.13.1. Greenness Index

This study provides an in‐depth assessment of the environmental impact of fluorescence‐based analytical methods and emphasizes the need for sustainable practices in the field of analytical chemistry [44]. We evaluated the environmental and safety aspects of our approach using an environmental performance evaluation framework and drew comparisons with already established techniques. Attention was paid to key experimental factors, such as the effective pH range of the method (2–12), together with a detailed analysis of solvent‐related hazards, including toxicity, corrosivity, and overall risk to the environment [45]. In contrast to traditional PR‐HPLC techniques, which primarily use acetonitrile, our method utilizes methanol as a solvent, representing a significant shift to safer and less toxic options.

Methanol helps to minimize both health risks and environmental impact, especially as there is no residual waste from the analysis. This approach is in line with the principles of green chemistry, which advocate the use of environmentally friendly substances and the reduction of waste at source. Figure 14(a) illustrates the method’s strong alignment with the core principles of environmentally sound analysis and highlights its suitability as a sustainable alternative to fluorescence‐based techniques. The data reflect growing potential for the integration of more environmentally friendly strategies into routine laboratory work. Prioritizing the use of environmentally friendly, less hazardous solvents contributes significantly to making research practice more environmentally conscious. These results also open up new avenues for the dissemination of environmentally friendly methods in FL applications and beyond, contributing not only to scientific progress but also to broader environmental protection goals. This novel method represents a significant advance in environmentally conscious analytical practice, as it improves both the speed of spectrofluorimetric reactions and the use of reagents, and offers better performance than conventional techniques for single‐agent analysis. With an AGREE score of 0.87, well above the benchmark of 0.6 for environmentally friendly analytical methods (Figure 14(b)), it demonstrates a strong commitment to environmental protection. The high score not only indicates a lower environmental impact, but also underlines the method’s ability to deliver efficient results without compromising analytical quality. With a focus on sustainability alongside performance, this approach reflects the growing movement toward more environmentally friendly laboratory practices and sets a precedent for the development of greener techniques in the future.

FIGURE 14(a) GAPA analysis and (b) AGREE score for the suggested PRM‐SDS‐alumina NP and PRM‐SDS‐NiO NP fluorescence method.

**Figure:**
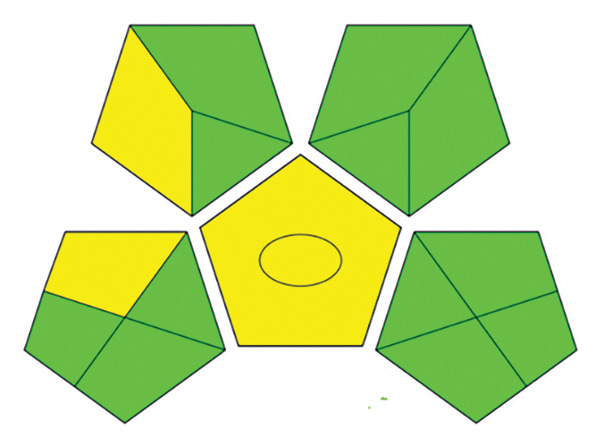
(a)

**Figure:**
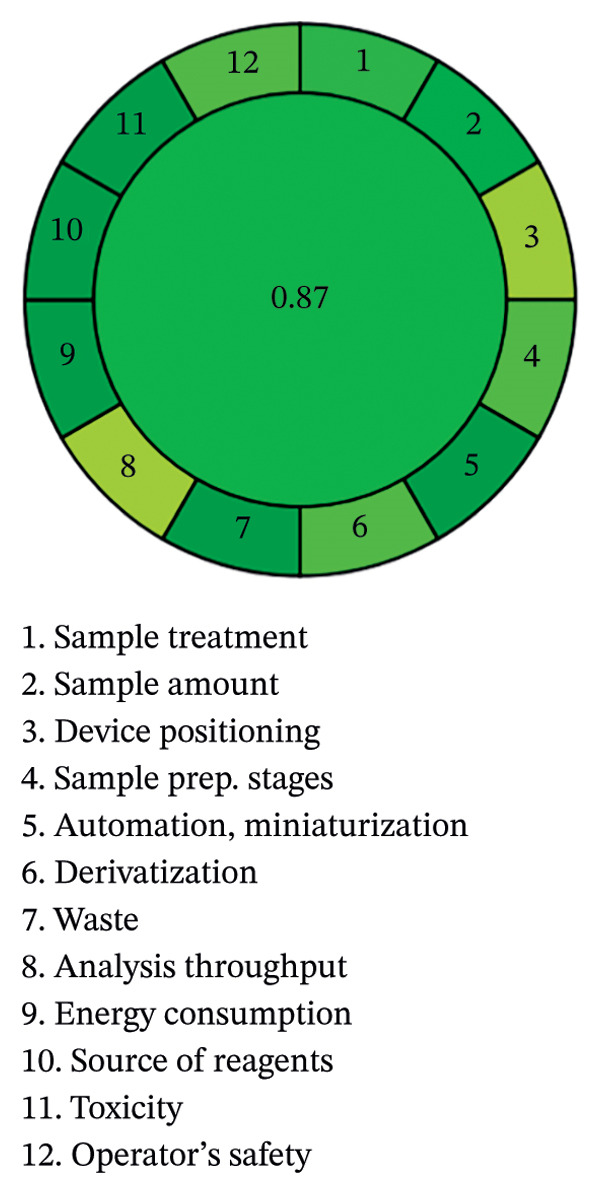
(b)

#### 3.13.2. Spider Chart Metric

To assess the sustainability profile of the chemicals in question, a multilevel radar chart was created containing five important assessment parameters: toxicity, physical properties, odor, flammability, and chemical stability. Each solvent was carefully evaluated and ranked on a scale from −5 (poor environmental performance) to +5 (high environmental performance). This analytical framework provides a comprehensive overview of the environmental impact of each solvent, considering various aspects of performance. The resulting visual representation provides a clear and concise comparison that highlights both the strengths and limitations of the solvents studied [46].

The spider diagrams provide a comprehensive visual analysis of the individual attributes associated with each assessment parameter and allow for easy comparison of solvent performance across multiple dimensions. In cases where the Safety Data Sheets (SDS) do not contain complete information, a neutral value of zero is assigned to indicate uncertainty and potential risks. To support this visual tool, there is a complementary “Environmental Compatibility Index Table” which lists the specific criteria assessed and indicates the percentage of data available for each solvent. This transparency helps to determine the confidence level of each assessment. By combining visual summaries with detailed data, the approach provides decision‐makers with valuable insight into the environmental and safety impacts of solvent selection, promoting informed, responsible decision‐making.

**FIGURE 15:**
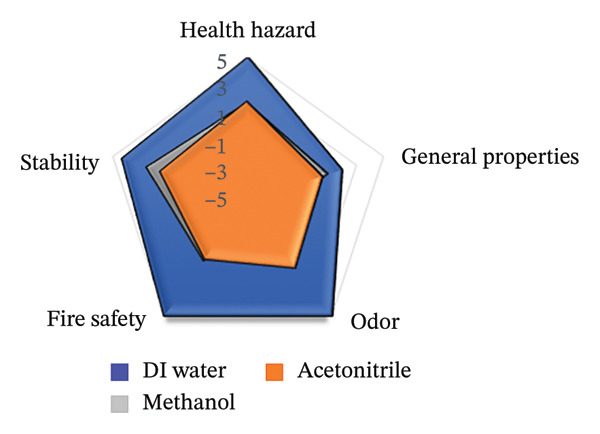
The spider chart compares the solvents used in the suggested spectrofluorimetric method with those used in chromatography and spectrophotometry methods.

This research introduces an innovative method for integrating environmental impact assessment into analytical chemistry, offering meaningful guidance for choosing appropriate solvents. As depicted in Figure 15, the main radar chart reveals that deionized water and ethanol exhibit significantly superior greenness scores relative to methanol and acetonitrile.

Supplementary spider diagrams provide a more in‐depth evaluation of the solvents, examining key factors, such as toxicity, physicochemical properties, stability, and flammability (Figures 16(a), 16(b), 16(c), 16(d)). In addition, Table 9 shows the environmental impact index, which summarizes both the average values and the proportion of data available for each solvent. The results underline the crucial role of selecting more environmentally friendly solvents in analytical methods and encourage the introduction of more sustainable methods in this area.

FIGURE 16Spider plots illustrating (a) health impact, (b) general properties, (c) stability, and (d) fire safety of the reagents utilized in the proposed spectrofluorimetric method, compared to those used in HPLC and spectrophotometric techniques.

**Figure:**
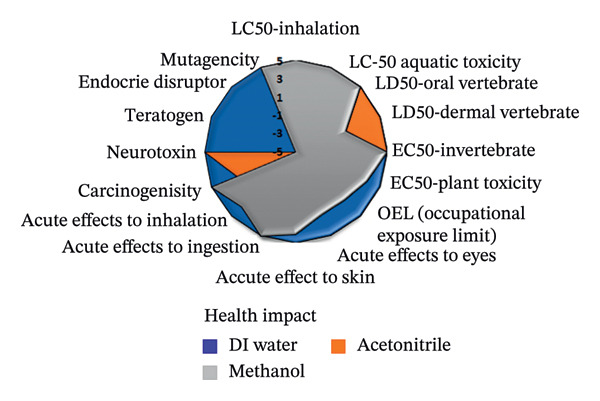
(a)

**Figure:**
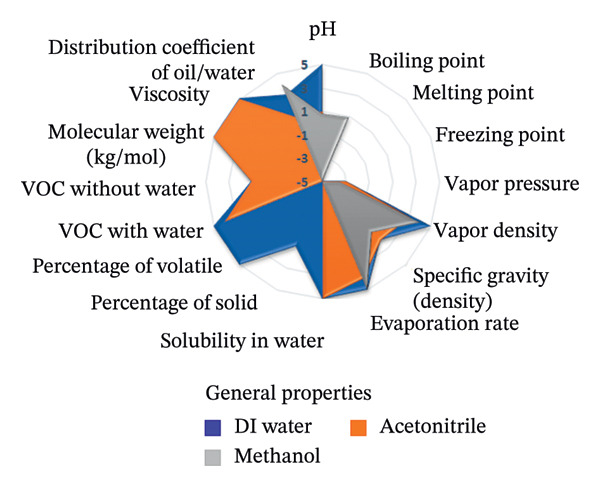
(b)

**Figure:**
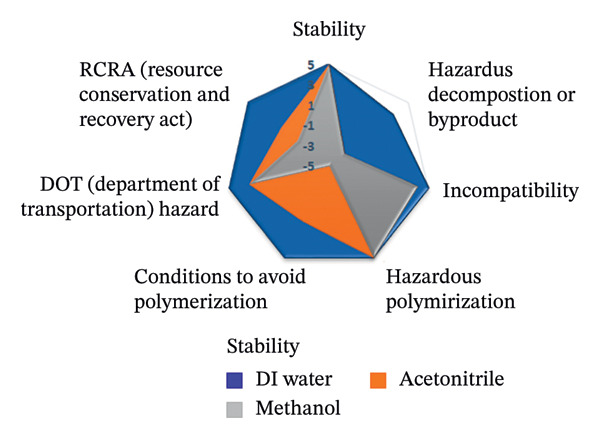
(c)

**Figure:**
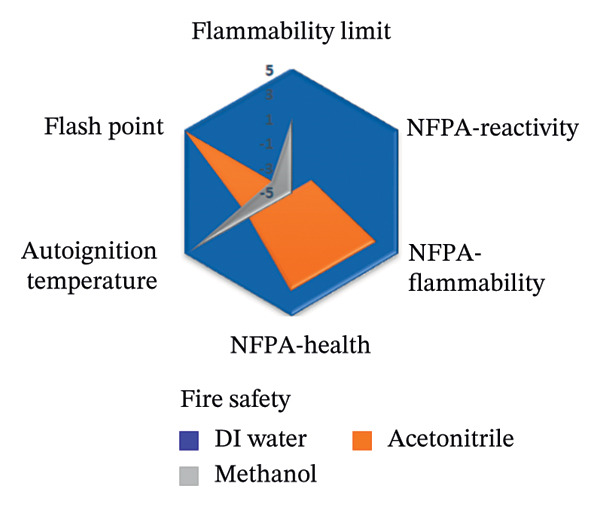
(d)

**TABLE 9:** Greenness index data for deionized water, methanol, and acetonitrile solvents.

Parameters	Water	Found information (%)	Methanol	Found information (%)	Acetonitrile	Found information (%)
Health impact	5	100	1.81	100	1.88	100
Stability	4.28	100	2.41	100	1.42	100
General properties	1.93	87.50	0.86	81.24	0.46	87.50
Fire safety	5	100	0.17	100	0.77	100
Odor	5	100	−3	100	0.00	100
Average	4.24	97.50	0.45	96.24	0.91	97.50

#### 3.13.3. Blueness Applicability Grade Index (BAGI)

This research paper presents an innovative approach to assessing the practical applicability of analytical techniques, the so‐called BAGI. The BAGI was developed with a focus on the practical principles of white analytical chemistry and serves as a supportive complement to the current green chemistry assessment frameworks. The index takes into account 10 key parameters that affect the practicality of a method, including the type of analysis, the number of analytes that can be measured in a single run, the speed of sample processing, the type of chemicals and materials used, instrument requirements, the ability to process multiple samples simultaneously, the degree of automation, sample preparation steps, and the amount of sample required for testing [47].

The efficacy of the proposed FL approach for the detection of PRM was evaluated and compared with three alternative analytical methods: spectrophotometric analysis, electrochemical techniques, and chromatographic separation methods. The evaluation was performed using the BAGI metric system, which produces two output formats, one in the form of a pictogram and the other as a numerical score. A visual scale with dark blue tones for a moderate match, lighter tones for a poor match and white for no match indicates how well the method meets the specified standards. The final score of the analysis method, which is between 25 and 100, is displayed as a number in the asteroid image of the BAGI pictogram. A score of 25 indicates minimum practicability, while a score of 100 indicates optimum practicability. To be considered “feasible,” a method should score at least 60 points, although this benchmark is recommended rather than mandatory.

The asteroid pictogram consists of 10 segments, each of which is linked to a specific performance criterion, and all of which are treated with the same meaning. The results show that each evaluated method fulfills the applicability requirements (Table 10). The BAGI for the proposed FL method was 82.5, indicating that it is very practical and suitable for analyzing the drug under study (Figures 17(a), 17(b), 17(c), 17(d)). The BAGI values for the different analytical methods show different degrees of performance. The second spectrophotometric method (82.5) outperforms the others, probably due to better calibration and optimization, and provides the highest accuracy and precision. The HPLC method (77.5) follows closely behind and has high reliability and precision, although it is slightly outperformed by the second spectrophotometric method. The first spectrophotometric method (75.0) has a lower performance, possibly due to limitations in sensitivity or resolution. The electrochemical method (72.5) performs the worst, indicating problems, such as lower sensitivity or difficulties in handling complex samples, making it the least effective in this comparison.

**TABLE 10:** The BAGI of the current spectrofluorimetric method for determination of PRM with respect to other analytical techniques.

No.	Analytical method	Parameters	BAGI score	Practical applicability
1.	RP‐HPLC [39]	Quantitative, single‐element analysis, HPLC instrument, samples (2–12), multistep preparation, 5‐10 samples/h, commercially available solvents, NO‐ precondition required, simple automated, <100 µL	77.5	Applicable
2.	Spectrophotometry [44]	Quantitative multielement analysis using UV detection; requires only a single sample with rapid preparation and analysis completed in under 1 hour. Utilizes commercially available reagents and manual operation, capable of processing more than 10 samples per hour.	75.0	Applicable
3.	Electrochemical method [48]	Quantitative single‐element analysis using a pH meter; involves one sample with a total analysis time of under 1 hour. Employs commercially available reagents, performed manually, and requires a sample volume of 10.1–50 mL.	72.5	Applicable
4.	Present work	Multielement investigation 2‐5 samples, simple and low‐cost sample preparation, common available solvents, fully automated analysis	82.5	Applicable

FIGURE 17BAGI pictogram: (a) HPLC, (b) spectrophotometry, (c) electrochemical, and (d) current spectrofluorimetric method for determination of PRM in different matrices (Current study spectrofluorimetry).

**Figure:**
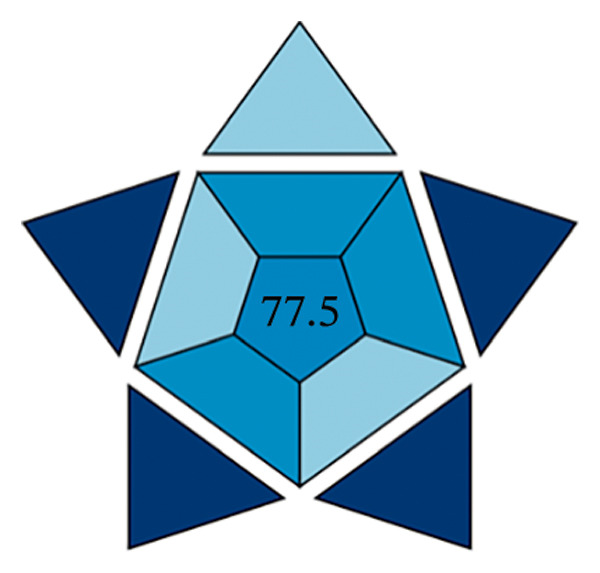
(a)

**Figure:**
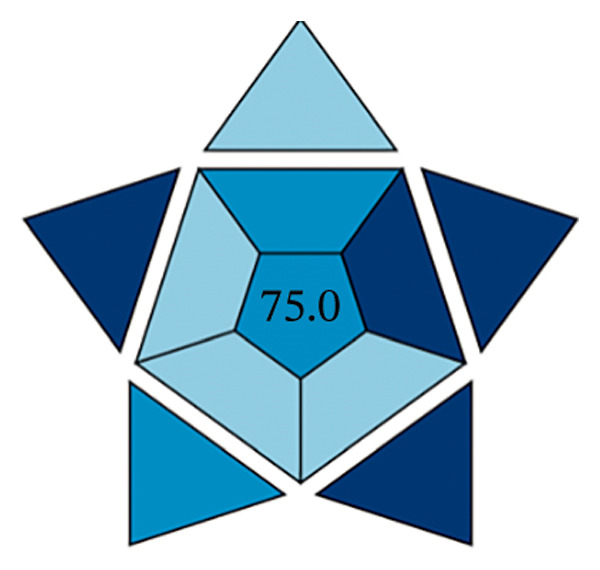
(b)

**Figure:**
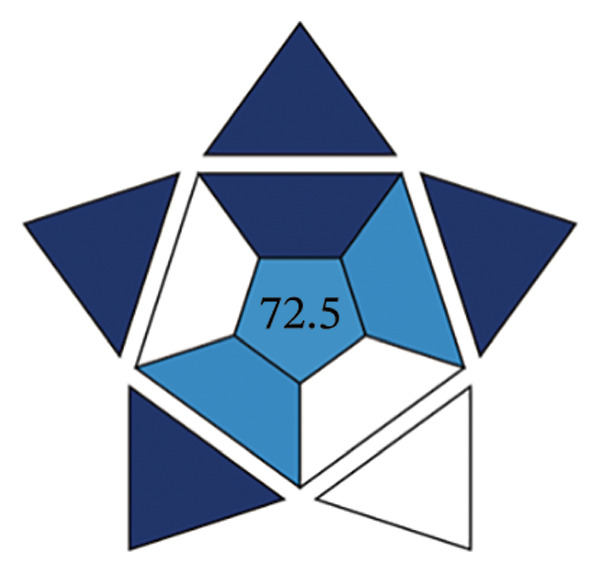
(c)

**Figure:**
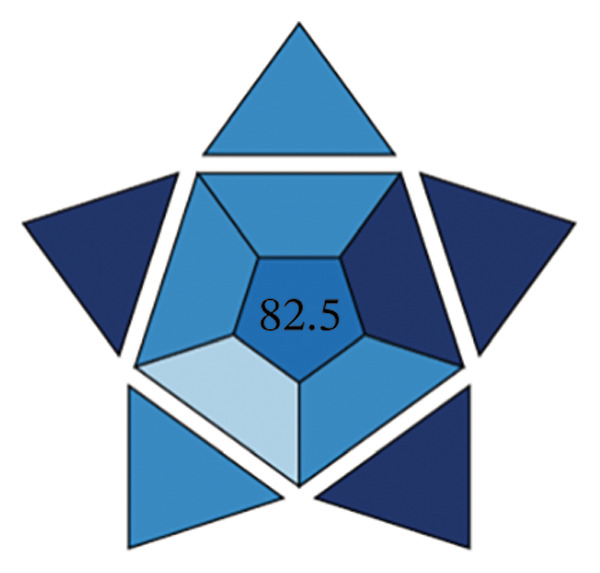
(d)

### 3.14. Advancement of the Proposed Method and the Limitations

In this study, we present a new, green spectrofluorimetric method for the sensitive detection of PRM using Shilajit‐derived metal oxide nanosensors, offering a sustainable and cost‐effective alternative to conventional analytical techniques. The innovative use of naturally sourced Shilajit as a precursor for nanosensor synthesis not only aligns with principles of green chemistry but also introduces a unique surface chemistry that enhances fluorescence response and detection sensitivity. This work advances the field of surface and interface science by elucidating the interaction mechanisms between drug molecules and nanostructured oxide surfaces, shedding light on how natural organic matrices can be transformed into functional nanosensors with tunable surface properties. The findings offer new insights into surface–analyte interactions and open pathways for ecofriendly sensor design in pharmaceutical analysis. However, limitations include potential batch‐to‐batch variability in Shilajit composition, which may affect reproducibility, and the need for further studies to validate the method across a broader range of pharmaceutical compounds and real‐world matrices.

## 4. Conclusion

Using plant extracts for NP synthesis offers significant environmental benefits compared to traditional chemical methods. This green synthesis approach utilizes natural, renewable resources rich in bioactive compounds that act as reducing and stabilizing agents, eliminating the need for hazardous chemicals and toxic solvents. As a result, it minimizes the generation of harmful byproducts and waste, reducing environmental pollution. Additionally, plant‐based synthesis often requires milder reaction conditions, such as lower temperatures and pressures, which leads to decreased energy consumption. Two innovative and straightforward fluorescence‐based methods for accurate quantification of PRM in both pure and dosed forms are presented using alumina and NiO NPs in combination with SDS as surfactant. By utilizing the unique optical properties of these nanomaterials, the methods successfully amplified fluorescent signals and achieved excellent recoveries of 99.07% ± 0.64% and 99.60% ± 0.37% for pure samples and 99.49% ± 0.45% and 99.54% ± 0.46% for tablet formulations. These results demonstrate the exceptional sensitivity and precision of the proposed methods, making them reliable and efficient tools for pharmaceutical analysis and drug monitoring. The integration of metal oxide NPs with SDS for fluorescence detection of PRM represents a significant advance over previous analytical techniques and provides a more effective approach for the determination of PRM. Conventional methods often struggle with lower sensitivity and longer processing times, which can hinder accurate and efficient analysis. However, by optimizing the fluorescence response and improving detection precision, this new method effectively overcomes these challenges. It creates a more reliable and consistent analytical framework that enables the detection of PRMs even at lower concentrations. This advance is particularly valuable for pharmaceutical applications as it supports more effective drug monitoring and quality control, ensuring better accuracy and faster results when analyzing pharmaceutical products. Unlike these older methods, which are time‐consuming and require specialized tools and preparation, spectrofluorimetry has the advantage of providing fast and accurate results with minimal preparation. Therefore, the method is ideal for scenarios that require fast turnaround, such as urgent clinical assessments or high‐throughput pharmaceutical production, where speed and reliability are critical. In addition, it represents a remarkable advance in the field of pharmaceutical analysis, as it offers enhanced capabilities that can be adapted to different environments, from academic research laboratories to hospital diagnostics. Its application supports more accurate drug monitoring and helps to raise the standards of pharmaceutical testing and therapeutic monitoring.

## Author Contributions

Azaa F. Al‐Shalawi formal analysis, methodology, and data curation; Amal M. Al‐Mohaimeed investigation, supervision, and conceptualization; Nawal A. Alarfaj supervision and visualization; Maha F. El‐Tohamy formal analysis, data curation, methodology, and writing original draft.

## Funding

The authors extend their appreciation to the Ongoing Research Funding Program (ORF‐2026‐247), King Saud University, Riyadh, Saudi Arabia, for financial support.

## Disclosure

All authors reviewed the final draft and provided their approval for its publication.

## Conflicts of Interest

The authors declare no conflicts of interest.

## Data Availability

Data supporting the results of this study can be obtained from the corresponding author upon reasonable request.
